# Well-posedness analysis and pseudo-Galerkin approximations using Tau Legendre algorithm for fractional systems of delay differential models regarding Hilfer (*α*,*β*)-framework set

**DOI:** 10.1371/journal.pone.0305259

**Published:** 2024-06-25

**Authors:** Hind Sweis, Omar Abu Arqub, Nabil Shawagfeh

**Affiliations:** 1 Department of Data Science, Faculty of Data Science, Arab American University, Ramallah, Palestine; 2 Department of Mathematics, Faculty of Science, Al-Balqa Applied University, Salt, Jordan; 3 Department of Mathematics, Faculty of Science, The University of Jordan, Amman, Jordan; National Research Centre, EGYPT

## Abstract

Fractional calculus serves as a versatile and potent tool for the modeling and control of intricate systems. This discussion debates the system of DFDEs with two regimes; theoretically and numerically. For theoretical analysis, we have established the EUE by leveraging the definition of Hilfer (*α*,*β*)-framework. Our investigation involved the examination of the possessions of the FRD, FCD, and FHD, utilizing their forcefulness and qualifications to convert the concerning delay system into an equivalent one of fractional DVIEs. By employing the CMT, we have successfully demonstrated the prescribed requirements. For numerical analysis, the Galerkin algorithm was implemented by leveraging OSLPs as a base function. This algorithm allows us to estimate the solution to the concerning system by transforming it into a series of algebraic equations. By employing the software MATHEMATICA 11, we have effortlessly demonstrated the requirements estimation of the nodal values. One of the key advantages of the deployed algorithm is its ability to achieve accurate results with fewer iterations compared to alternative methods. To validate the effectiveness and precision of our analysis, we conducted a comprehensive evaluation through various linear and nonlinear numerical applications. The results of these tests, accompanied by figures and tables, further support the superiority of our algorithm. Finally, an analysis of the numerical algorithm employed was provided along with insightful suggestions for potential future research directions.

## 1 Foundations

Fractional calculus is a discipline within modern mathematics and computational physics that focuses on studying derivatives and integrals with fractional orders. While initially introduced by Leibniz in the late 17th century, it did not gain significant attention until the late 20th century. This can be partly attributed to advancements in computing technology during this period, as they facilitated efficient solutions to fractional models [1–3]. The applications of fractional patterns span various domains including engineering, signal processing, statistics, and others. For instance, fractional calculus finds utility in modeling the behavior of viscoelastic materials, controlling robotic systems, predicting financial markets, and modeling biological systems [4–7].

The DFDEs are a class of ordinary calculus that emphasizes fractional derivatives and delays. The scope of DFDEs is more comprehensive than traditional delay models, allowing for a broader range of phenomena to be represented. Applications of DFDEs appear in diverse domains involving signal processing, statistics, engineering, and biology where various simulation techniques are used [8–19]. For instance, in physics, DFDEs have been used to model heat transfer, diffusion, and wave propagation in materials with memory properties [20, 21]. In engineering, DFDEs have proven useful for modeling and controlling systems such as robots, aircraft, and power networks [7, 22–24]. Within biology, DFDEs have been employed to formulate agent-based representations of systems like the cardiovascular, nervous, and immune systems [25, 26].

The DFDE is an equation in which the derivative of a function, ϑ_ϐ_(ƻ) with ϐ = 1,2, at a certain time, ƻ, involves values of the function, *ϑ*_*ϐ*_(ƻ−σ_ϐ_) with ϐ = 1,2, at a previous time, ƻ−σ_ϐ_ with ϐ = 1,2. The simplest constant delay equation takes the form $\vartheta_{ϐ}^{\prime }(ƻ)=S_{ϐ}(ƻ,{℘}_{ϐ}(\vartheta_{1}(ƻ),\vartheta_{2}(ƻ)),\vartheta_{ϐ}(ƻ-\sigma_{ϐ}))$ with ϐ = 1,2, where *ϑ*_*ϐ*_(ƻ−σ_ϐ_) with ϐ = 1,2 representing the value of ϑ_ϐ_ with ϐ = 1,2 at a constant time σ_ϐ_ with ϐ = 1,2 units in the past, making the effect *ϑ*_*ϐ*_ with ϐ = 1,2 on the current rate of change of ϑ_ϐ_ delayed by a time σ_ϐ_ with ϐ = 1,2.

This paper presents the necessary conditions for the EUE together with numerical simulation concerning the resulting set of DFDEs

$${\;}^{H}D_{0}^{\alpha ,\beta }\vartheta_{ϐ}(ƻ)=S_{ϐ}(ƻ,{℘}_{ϐ}(\vartheta_{1}(ƻ),\vartheta_{2}(ƻ)),\vartheta_{ϐ}(ƻ-\sigma_{ϐ})),ϐ=1,2,$$ (1)

accordance with the constraints

$$\left\{ \begin{array}{l} {\vartheta_{ϐ}(ƻ)=\varphi }_{ϐ}(ƻ),-\sigma_{ϐ}<ƻ\leq 0,\\ I_{0^{+}}^{1-\gamma }\varphi_{ϐ}(0^{+})=\rho_{ϐ}. \end{array}\right.$$ (2)

Herein, ƻ∈ *J*: = [0,ℱ] with ℱ>0,*α*∈(0,1) and *β*∈[0,1] providing *α*<*γ*<1 with $\gamma =(1-\beta )(\alpha -1)+1,\rho_{ϐ}\in R,\varphi_{ϐ}\in L^{\frac{1}{p}}(J),p\in (0,\frac{1-\gamma +\alpha }{2}),\sigma_{ϐ}>0,S_{ϐ}\in C(J\times R^{2}\to R),{℘}_{ϐ}\in (R^{2}\to R)$, and ${\;}^{H}D_{0}^{\alpha ,\beta }\vartheta_{ϐ}$ relate to the FHD [27–31] of *ϑ*_*ϐ*_ of fraction *α* and type *β*.

Our primary concern is to obtain a quantitative estimate of (1) through examination of the elegances of the SGLA, which depends on orthogonal spline basis functions [32–36]. Previous investigations have employed the presented scheme to approximate various differential or integral problems, a few of which will be mentioned. In [36], researchers successfully characterized the telegraph fraction model leveraging the SGLA. Similarly, in [35], the SGLA was utilized to find approximation of Fredholm fraction integrodifferential problems.

In this particular research, we adopted the parameterization of OSLPs to substitute the necessary background functions in (1). Subsequently, the SGLA was employed to convert (1) into sets of algebraic equations. Upon handling the producing set, the desired approximate solution was obtained. One notable numerical optimality of the SGLA employed is its feasibility for any model formalism. Furthermore, it enumerates significantly valid approximations by leveraging a minimal number of OSLP terms.

The organization of the computations and algorithm development are ordered as next. Section 2 presents spectrum attributes, and necessary lemmas based on the definitions of FRD, FCD, and FHD. In Section 3, we transform the system described in (1) and (2) subjected to a congruent set of DVIEs. Additionally, this section outlines the development of proof for the EUE concerning (1) and (2). In Section 4, we introduce the SGLA as a solution technique for tackling (1) and (2) and proceed to characterize the theorems on convergence. Section 5 provides numerical applications and results. Finally, a summary of key findings is mentioned in Section 6.

## 2 Background and overview results

This section presents indispensable background and properties associated with FRD, FCD, and FHD approaches, simultaneously, with significant outcomes that will be exploited in the next portion. Anyhow, consider the following requirements:

- The Banach topological manifold *L*^*P*^(*J*,ℝ) is axiomatized as the collection of each Lebesgue measurable S:J→R equipped with ${\left\|S\right\|}_{L^{p}}={\left(\int_{J}{\left|S(ƻ)\right)}^{p}dƻ\right)}^{\frac{1}{p}}<\infty$.
- The topological manifold AC(J,R)={S:J→R;SabsolutelycontinuousonJ}. Indeed, the topological manifold ${AC}^{\hbar }(J,R)=\{S:S\in C^{\hbar -1}(J,R)\;and\;S^{\hbar -1}\in AC(J,R)\}$.
- The weighted topological manifold $C_{\theta }(J)=\{S:J\to R;{ƻ}^{\theta }S(ƻ)\in C(J,R)\}$ with ${\left\|S\right\|}_{C_{\theta }}=\underset{ƻ\in J}{\max}{ƻ}^{\theta }|S(ƻ)|$.

**Definition 1.** [37] The left-side integral of fraction *θ*>0 for $S\in L^{1}(J)$ is

$$(I_{0^{+}}^{\theta }S)(ƻ)=\frac{1}{\Gamma (\theta )}\int_{0}^{ƻ}{\left(ƻ-s\right)}^{\theta -1}S(s)ds.$$ (3)

**Definition 2.** [37] If $S(ƻ)\in {AC}^{\hbar }(J,R)$, then the left-side FRD of fraction *θ*∈(ℏ−1,ℏ) with ℏ∈ℕ exists a.e. on *J* with

$$({\;}^{L}D_{0^{+}}^{\theta }S)(ƻ)=\frac{1}{\Gamma (\hbar -\theta )}\frac{d^{\hbar }}{d{ƻ}^{\hbar }}\int_{0}^{ƻ}‍{\left(ƻ-s\right)}^{\hbar -\theta -1}S(s)ds.$$ (4)

**Remark 1.** If *θ*>0 and $\hat{\theta }>0$, then

$$[I_{0^{+}}^{\theta }s^{\hat{\theta }-1}](ƻ)=\frac{\Gamma (\hat{\theta })}{\Gamma (\hat{\theta }+\theta )}{ƻ}^{\hat{\theta }+\theta -1}.$$

$$[{\;}^{L}D_{0^{+}}^{\theta }s^{\hat{\theta }-1}](ƻ)=\frac{\Gamma (\hat{\theta })}{\Gamma (\hat{\theta }-\theta )}{ƻ}^{\hat{\theta }-\theta -1}.$$

$$[{\;}^{L}D_{0^{+}}^{\theta }s^{\theta -ϐ}](ƻ)=0,ϐ=1,2,\cdots ,[\theta ]+1.$$

**Definition 3.** [38] The FCD of fraction *θ*∈(ℏ−1,ℏ) with ℏ∈ℕ is

$${\;}^{C}D_{0}^{\theta }S(ƻ)=\frac{1}{\Gamma (\hbar -\theta )}\int_{0}^{ƻ}\frac{S^{\left(\hbar \right)}(s)}{{\left(ƻ-s\right)}^{\theta +1-\hbar }}ds.$$ (5)

**Definition 4.** [27] Let $D=\frac{d}{dƻ}$ and *γ* = *α*+ℏ*β*−*αβ*. The left-side FHD of fraction *α*∈(ℏ−1,ℏ) with ℏ∈ℕ and *β*∈[0,1] of S is

$$({\;}^{H}D_{0^{+}}^{\alpha ,\beta }S)(ƻ)=(I_{0^{+}}^{\beta (\hbar -\alpha )}D^{\hbar }I_{0^{+}}^{\left(1-\beta )(\hbar -\alpha \right)}S)(ƻ)=(I_{0^{+}}^{\gamma -\alpha }D^{\hbar }I_{0^{+}}^{\hbar -\gamma }S)(ƻ),ƻ\in J.$$ (6)

The term ${\;}^{H}D_{0^{+}}^{\alpha ,\beta }$ can be formatted as ${\;}^{H}D_{0^{+}}^{\alpha ,\beta }=I_{0^{+}}^{\beta (\hbar -\alpha )}D^{\hbar }I_{0^{+}}^{\left(1-\beta )(\hbar -\alpha \right)}=I_{0^{+}}^{\beta (\hbar -\alpha )}{{\;}^{L}D}_{0^{+}}^{\gamma }=I_{0^{+}}^{\left(\gamma -\alpha \right)}{{\;}^{L}D}_{0^{+}}^{\gamma }$. Indeed, if *β* = 0, then the left-side FRD is ${{\;}^{L}D}_{0^{+}}^{\alpha }={{\;}^{H}D}_{0^{+}}^{\alpha ,0}$ and if *β* = 1, then the left-side FCD is ${{\;}^{C}D}_{0^{+}}^{\alpha }={{\;}^{H}D}_{0^{+}}^{\alpha ,1}.$

**Lemma 1.** [28] The fundamental solution of ${\;}^{H}D_{0^{+}}^{\alpha ,\beta }S(ƻ)=0$ is $S(ƻ)=\sum_{ϐ=1}^{\hbar }c_{ϐ}{\left(ƻ-0\right)}^{\alpha +\hbar \beta -\alpha \beta -ϐ}$ with ƻ>0,α+ℏβ−αβ∈(ℏ−1,ℏ), and $c_{ϐ}\in R$.

**Lemma 2.** [28] Let *ρ*∈(0,1), υ∈(−1,0], and *ρ*+*υ*>0, then at $0<p<\frac{\rho +\upsilon }{2}$ and $\vartheta \in L^{\frac{1}{p}}$, one has $\left(I_{0^{+}}^{\rho }s^{\upsilon }\vartheta (s))(ƻ)\in AC(J,R\right)$.

**Lemma 3.** [28] Let *θ*∈(ℏ−1,ℏ) with ℏ∈ℕ. If *ϑ*∈*L*^1^(*J*) and $I_{0^{+}}^{\hbar -\theta }\vartheta \in AC^{\hbar }(J)$, then

$${(I}_{0^{+}}^{\theta }{\;}^{L}D_{0^{+}}^{\theta }\vartheta )(ƻ)=\vartheta (ƻ)-\sum_{ϐ=1}^{\hbar }\frac{{{(I}_{0^{+}}^{\hbar -\theta }\vartheta )}^{\left(\hbar -ϐ\right)}(0^{+})}{\Gamma (\theta -ϐ+1)}{ƻ}^{\theta -ϐ}.$$ (7)

**Lemma 4.** [31] Let *ϑ*∈*L*^1^(*J*) and ${\;}^{L}D_{0^{+}}^{\gamma -\alpha }\vartheta \in L^{1}(J)$ exists. Then $({\;}^{H}D_{0^{+}}^{\alpha ,\beta }I_{0^{+}}^{\alpha }\vartheta )(ƻ)=I_{0^{+}}^{\gamma -\alpha }{\;}^{L}D_{0^{+}}^{\gamma -\alpha }\vartheta (ƻ)$.

**Lemma 5.** [31] Let *α*>0 and 0≤*γ*<1. Then $I_{0^{+}}^{\alpha }$ is bounded from *C*_*γ*_(*J*) into *C*_*γ*_(*J*).

Here, we define the following topological manifold: $C_{1-\gamma }^{\alpha ,\beta }(J)=\{S\in C_{1-\gamma }(J);({{\;}^{H}D}_{0^{+}}^{\alpha ,\beta }S)(ƻ)\in C_{1-\gamma }(J)\}$ and $C_{1-\gamma }^{\gamma }(J)=\{S\in C_{1-\gamma }(J);({{\;}^{L}D}_{0^{+}}^{\gamma }S)(ƻ)\in C_{1-\gamma }(J)\}.$ Note that ${{\;}^{H}D}_{0^{+}}^{\alpha ,\beta }℘=I_{0^{+}}^{\beta (1-\alpha )}{\;}^{L}D_{0^{+}}^{\gamma }℘$, so, by Lemma 6 $C_{1-\gamma }^{\gamma }(J)\subset C_{1-\gamma }^{\alpha ,\beta }(J)$.

**Lemma 6.** [31] Let $℘\in L^{1}(0,c)$ and *θ*>0. Then

$$\underset{ƻ\to c^{+}}{\lim}\int_{0}^{c}{\left(ƻ-s\right)}^{\theta -1}℘(s)ds=\int_{0}^{c}{\left(c-s\right)}^{\theta -1}℘(s)ds=\Gamma (\theta )I_{0^{+}}^{\theta }℘(c).$$ (8)

**Lemma 7.** [31] Let $\theta \in (0,1),0<c<F,℘\in C_{\theta }[0,c]$, ℘∈C[c,F], and ℘ is continuous at c. Then ℘∈*C*_*θ*_(*J*).

## 3 Congruence fractional DVIE and the results concerning EUE

In this section, we investigate the examination of the possessions of the FRD, FCD, and FHD utilizing their forcefulness and qualifications to convert the concerning delay system into an equivalent one of fractional DVIEs. After that, we visualize and clarify the EUE concerning (1) and (2) leveraging its congruence system of fractional DVIEs (9) within $C_{1-\gamma }^{\gamma }(J)$.

**Theorem 1.** Let $0<p<\frac{1-\gamma +\alpha }{2},℘\in C_{1-\gamma }(R,R),\vartheta_{1},\vartheta_{2}\in C_{1-\gamma }(J,R)$, and $S_{1},S_{2}:J\times R^{2}\times \to R$ be as $S_{1}(∙,℘(\vartheta (∙)),\vartheta_{1}(∙-\sigma_{1}))\in L^{\frac{1}{p}}(J,R,R)$ and $S_{2}(∙,℘(\vartheta (∙)),\vartheta_{2}(∙-\sigma_{2}))\in L^{\frac{1}{p}}(J,R,R)$. Then *ϑ*(ƻ) fulfills Problem (1) and (2) iff *ϑ*(ƻ) fulfills

$$\vartheta_{ϐ}(ƻ)=\frac{\rho_{ϐ}{ƻ}^{\gamma -1}}{\Gamma (\gamma )}+\int_{0}^{ƻ}\frac{{\left(ƻ-s\right)}^{\alpha -1}S_{ϐ}(s,{℘}_{ϐ}(\vartheta (s)),\vartheta_{ϐ}(s-\sigma_{ϐ}))}{\Gamma (\alpha )}ds,ϐ=1,2.$$ (9)

**Proof.** For ƻ∈*J* and γ−α∈(0,1−α) it suffices from Lemma 2 at ρ=1−(γ−α) and υ=0 that $1-(\gamma -\alpha )>0,0<p<\frac{1-(\gamma -\alpha )}{2}$, and $S_{1},S_{2}\in L^{\frac{1}{p}}$. So $I_{0^{+}}^{1-(\gamma -\alpha )}S_{ϐ}(s,{℘}_{ϐ}(\vartheta (s)),\vartheta_{ϐ}(s-\sigma_{ϐ}))(ƻ)\in AC(J\times R^{2},R)$ and ${\;}^{L}D_{0^{+}}^{\alpha }S_{ϐ}\in L^{1}(J\times R^{3})$.

From Lemma 3, take *θ* = *γ*−*α*, one gained

$$(I_{0^{+}}^{\gamma -\alpha }{\;}^{L}D_{0^{+}}^{\gamma -\alpha }S_{ϐ}(s,{℘}_{ϐ}(\vartheta (s)),\vartheta_{ϐ}(s-\sigma_{ϐ})))(ƻ)=S_{ϐ}(s,{℘}_{ϐ}(\vartheta (s)),\vartheta_{ϐ}(s-\sigma_{ϐ}))-\frac{{(I}_{0^{+}}^{1-(\gamma -\alpha )}S_{ϐ}(s,{℘}_{ϐ}(\vartheta (s)),\vartheta_{ϐ}(s-\sigma_{ϐ}))(0^{+})}{\Gamma (\gamma -\alpha )}{ƻ}^{\gamma -\alpha -1}.$$ (10)

From Lemma 4, one concludes

$$I_{0^{+}}^{\gamma -\alpha }{\;}^{L}D_{0^{+}}^{\gamma -\alpha }S_{ϐ}(s,{℘}_{ϐ}(\vartheta (s)),\vartheta_{ϐ}(s-\sigma_{ϐ}))(ƻ)={\;}^{H}D_{0^{+}}^{\alpha ,\beta }I_{0^{+}}^{\alpha }(S_{ϐ}(s,{℘}_{ϐ}(\vartheta (s)),\vartheta_{ϐ}(s-\sigma_{ϐ})))(ƻ).$$ (11)

Then

$${\;}^{H}D_{0^{+}}^{\alpha ,\beta }({\vartheta_{ϐ}(ƻ)-I}_{0^{+}}^{\alpha }S_{ϐ}(s,{℘}_{ϐ}(\vartheta (s)),\vartheta_{ϐ}(s-\sigma_{ϐ}))(ƻ))=S_{ϐ}(s,{℘}_{ϐ}(\vartheta (s)),\vartheta_{ϐ}(s-\sigma_{ϐ}))-S_{ϐ}(s,{℘}_{ϐ}(\vartheta (s)),\vartheta_{ϐ}(s-\sigma_{ϐ}))+\frac{\left(I_{0^{+}}^{1-(\gamma -\alpha )}S_{ϐ}(s,{℘}_{ϐ}(\vartheta (s)),\vartheta_{ϐ}(s-\sigma_{ϐ})))(0^{+}\right)}{\Gamma (\gamma -\alpha )}{ƻ}^{\gamma -\alpha -1}.$$ (12)

Since $(I_{0^{+}}^{1-(\gamma -\alpha )}S_{ϐ}(s,{℘}_{ϐ}(\vartheta (s)),\vartheta_{ϐ}(s-\sigma_{ϐ})))(0^{+})=0.$ So, ${\;}^{H}D_{0^{+}}^{\alpha ,\beta }(\vartheta_{ϐ}(ƻ)-(I_{0^{+}}^{\alpha }S_{ϐ}(s,{℘}_{ϐ}(\vartheta (s)),\vartheta_{ϐ}(s-\sigma_{ϐ})))(ƻ))=0$. Using Lemma 1, one concludes whenever $c_{ϐ}\in R$ that

$$\vartheta_{ϐ}(ƻ)=c_{ϐ}{ƻ}^{\gamma -1}+(I_{0^{+}}^{\alpha }S_{ϐ}(s,{℘}_{ϐ}(\vartheta (s)),\vartheta_{ϐ}(s-\sigma_{ϐ})))(ƻ),ƻ>0.$$ (13)

Using ${\vartheta_{ϐ}(ƻ)=\varphi }_{ϐ}(ƻ)$ with $-\sigma_{ϐ}<ƻ\leq 0$ such that $I_{0^{+}}^{1-\gamma }\varphi_{ϐ}(0^{+})=\rho_{ϐ}$ and $I_{0^{+}}^{1-\gamma }\vartheta_{ϐ}(0^{+})=c_{ϐ}\Gamma (\gamma )+0,-\sigma_{ϐ}\leq ƻ\leq 0$, we have $c_{ϐ}\Gamma (\gamma )=\rho_{ϐ}$. So the fractional DVIEs are

$$\vartheta_{ϐ}(ƻ)=\frac{\rho_{ϐ}{ƻ}^{\gamma -1}}{\Gamma (\gamma )}+(I_{0^{+}}^{\alpha }S_{ϐ}(s,{℘}_{ϐ}(\vartheta (s)),\vartheta_{ϐ}(s-\sigma_{ϐ})))(ƻ),ϐ=1,2.$$ (14)

In the consecutive results, $\vartheta (ƻ)=(\vartheta_{1}(ƻ),\vartheta_{2}(ƻ)),\tilde{\vartheta }(ƻ)=({\tilde{\vartheta }}_{1}(ƻ),{\tilde{\vartheta }}_{2}(ƻ)),{\;}^{H}D_{0}^{\alpha ,\beta }\vartheta (ƻ)=({\;}^{H}D_{0}^{\alpha ,\beta }\vartheta_{1}(ƻ),{\;}^{H}D_{0}^{\alpha ,\beta }\vartheta_{2}(ƻ))$, and $\vartheta (ƻ-\theta )=(\vartheta_{1}(ƻ-\sigma_{1}),\vartheta_{2}(ƻ-\sigma_{2}))$with *θ* = (*σ*_1_, *σ*_2_). Indeed, $℘(\vartheta (ƻ))=({℘}_{1}(\vartheta (ƻ)),{℘}_{2}(\vartheta (ƻ)))$ and $S(ƻ,℘(\vartheta (ƻ)),\vartheta (ƻ-\theta ))=(S_{1}(ƻ,{℘}_{1}(\vartheta (ƻ)),\vartheta_{1}(ƻ-\sigma_{1})),S_{2}(ƻ,{℘}_{2}(\vartheta (ƻ)),\vartheta_{2}(ƻ-\sigma_{2})))$.

**Theorem 2.** For any $\vartheta :J\to R^{2},℘\in C_{1-\gamma }(R^{2},R),$ and $\vartheta (ƻ-\theta )\in C_{1-\gamma }(J)$, if S fulfills the Lipschitzian

$${\left\|S(ƻ,℘(\vartheta (ƻ)),\vartheta (ƻ-\theta ))-S(ƻ,℘(\tilde{\vartheta }(ƻ)),\tilde{\vartheta }(ƻ-\theta ))\right\|}_{C_{1-\gamma }(J)}\leq L({\left\|\vartheta (ƻ)-\tilde{\vartheta }(ƻ)\right\|}_{C_{1-\gamma }(J)}+{\left\|\vartheta (ƻ-\theta )-\tilde{\vartheta }(ƻ-\theta )\right\|}_{C_{1-\gamma }(J)}),$$ (15)

then ∃!-Sol for (1) and (2) in $C_{1-\gamma }^{\gamma }(J)$.

**Proof.** Initially, we prove the EUE within *C*_1−*γ*_(*J*). Our demonstration relies on the examination of three scenarios: the first encompasses ƻ∈(0,*σ*_1_], the second encompasses ƻ∈(*σ*_1_, *σ*_2_], and the third one encompasses ƻ∈(*σ*_2_,ℱ]. In each scenario, we divide the *J* into subintervals and validate *G*: *S*→*S* defined as

$$G\vartheta (ƻ)=(G\vartheta_{1}(ƻ),G\vartheta_{2}(ƻ))$$ (16)

$$=\left(\frac{\rho_{1}{ƻ}^{\gamma -1}}{\Gamma (\gamma )}+\int_{0}^{ƻ}\frac{{\left(ƻ-s\right)}^{\alpha -1}S_{1}(s,{℘}_{1}(\vartheta (s)),\vartheta_{1}(s-\sigma_{1}))}{\Gamma (\alpha )}ds,\frac{\rho_{2}{ƻ}^{\gamma -1}}{\Gamma (\gamma )}+\int_{0}^{ƻ}\frac{{\left(ƻ-s\right)}^{\alpha -1}S_{2}(s,{℘}_{2}(\vartheta (s)),\vartheta_{2}(s-\sigma_{2}))}{\Gamma (\alpha )}ds\right),$$

where $S=\{\vartheta \in C_{1-\gamma }(J)\}$ is a contraction mapping.

Assuming that $\sigma_{1}\leq \sigma_{2}$. Then we consider three cases as follows:

**Case 1.** Let ƻ∈(0,*σ*_1_], so $\vartheta (ƻ-\theta )=\tilde{\vartheta }(ƻ-\theta )=\varphi (ƻ-\theta )=(\varphi_{1}(ƻ-\sigma_{1}),\varphi_{2}(ƻ-\sigma_{2}))$ and choose $c_{1},c_{2}$ such that $0<c_{1}\leq c_{2}\leq \sigma_{1}.\;The\;metric\;space\;C_{1-\gamma }[c_{1},c_{2}]$ is considered complete with

$$d(\vartheta (ƻ),\tilde{\vartheta }(ƻ))={\left\|\vartheta (ƻ)-\tilde{\vartheta }(ƻ)\right\|}_{C_{1-\gamma }[c_{1},c_{2}]}=\underset{ƻ\in [c_{1},c_{2}]}{\max}\left\|{ƻ}^{1-\gamma }(\vartheta (ƻ)-\tilde{\vartheta }(ƻ))\right\|.$$ (17)

Select ${ƻ}_{1}\in (0,\sigma_{1}]$ and note that $\frac{\rho_{1}}{\Gamma (\gamma )}{ƻ}^{\gamma -1},\frac{\rho_{2}}{\Gamma (\gamma )}{ƻ}^{\gamma -1}\in C_{1-\gamma }[0,{ƻ}_{1}]$, so by Lemma 5 due to $G\vartheta \in C_{1-\gamma }[0,{ƻ}_{1}]$, thus G maps $C_{1-\gamma }[0,{ƻ}_{1}]$ into itself. Now, $\forall \vartheta (ƻ),\tilde{\vartheta }(ƻ)\in C_{1-\gamma }[0,{ƻ}_{1}]$, we have

$${\left\|G\vartheta (ƻ)-G\tilde{\vartheta }(ƻ)\right\|}_{C_{1-\gamma }[0,{ƻ}_{1}]}\leq |\frac{\left(\rho_{1}-\rho_{2}\right)}{\Gamma (\gamma )}|{\left\|I_{0^{+}}^{\alpha }|S(ƻ,℘(\vartheta (ƻ)),\vartheta (ƻ-\theta ))-S(ƻ,℘(\tilde{\vartheta }(ƻ)),\tilde{\vartheta }(ƻ-\theta ))|\right\|}_{C_{1-\gamma }[0,{ƻ}_{1}]}.$$ (18)

Note that

$${\left\|S(ƻ,℘(\vartheta (ƻ)),\vartheta (ƻ-\theta ))-S(ƻ,℘(\tilde{\vartheta }(ƻ)),\tilde{\vartheta }(ƻ-\theta ))\right\|}_{C_{1-\gamma }[0,{ƻ}_{1}]}=\underset{ƻ\in [0,{ƻ}_{1}]}{\max}\left\|{ƻ}^{1-\gamma }(S(ƻ,℘(\vartheta (ƻ)),\vartheta (ƻ-\theta ))-S(ƻ,℘(\tilde{\vartheta }(ƻ)),\tilde{\vartheta }(ƻ-\theta )))\right\|.$$ (19)

So,

$$|S(ƻ,℘(\vartheta (ƻ)),\vartheta (ƻ-\theta )-S(ƻ,℘(\tilde{\vartheta }(ƻ)),\tilde{\vartheta }(ƻ-\theta ))|\leq {ƻ}^{\gamma -1}{\left\|S(ƻ,℘(\vartheta (ƻ)),\vartheta (ƻ-\theta ))-S(ƻ,℘(\tilde{\vartheta }(ƻ)),\tilde{\vartheta }(ƻ-\theta ))\right\|}_{C_{1-\gamma }[0,{ƻ}_{1}]}.$$ (20)

$${\left\|G\vartheta (ƻ)-G\tilde{\vartheta }(ƻ)\right\|}_{C_{1-\gamma }[0,{ƻ}_{1}]}$$

$$\leq \frac{\left|\rho_{1}-\rho_{2}\right|}{\Gamma (\gamma )}\frac{\Gamma (\gamma )}{\Gamma (\gamma +\alpha )}{{ƻ}_{1}}^{\gamma +\alpha -1}L({\left\|\vartheta (ƻ)-\tilde{\vartheta }(ƻ)\right\|}_{C_{1-\gamma }[0,{ƻ}_{1}]}+{\left\|\vartheta (ƻ-\theta )-\tilde{\vartheta }(ƻ-\theta )\right\|}_{C_{1-\gamma }[0,{ƻ}_{1}]})$$

$$=\frac{\left|\rho_{1}-\rho_{2}\right|}{\Gamma (\gamma +\alpha )}{{ƻ}_{1}}^{\gamma +\alpha -1}L({\left\|\vartheta (ƻ)-\tilde{\vartheta }(ƻ)\right\|}_{C_{1-\gamma }[0,{ƻ}_{1}]}+{\left\|\varphi (ƻ-\theta )-\varphi (ƻ-\theta )\right\|}_{C_{1-\gamma }[0,{ƻ}_{1}]})$$

$$=\mu_{1}{\left\|\vartheta (ƻ)-\tilde{\vartheta }(ƻ)\right\|}_{C_{1-\gamma }[0,{ƻ}_{1}]}.$$ (21)

where $\mu_{1}=\frac{L|\rho_{1}-\rho_{2}|}{\Gamma (\gamma +\alpha )}{{ƻ}_{1}}^{\gamma +\alpha -1}<1$.

Given that *μ*_1_<1, the CMT guarantees the existence of a single fixed point, denoted as $\vartheta_{0}(ƻ)\in C_{1-\gamma }[0,{ƻ}_{1}]$ within (0,ƻ_1_]. In the case where ƻ_1_≠*σ*_1_, we examine [ƻ_1_, *σ*_1_]. It is important to note that

$$G\vartheta (ƻ)=\left(\frac{\rho_{1}{ƻ}^{\gamma -1}}{\Gamma (\gamma )}+\int_{0}^{ƻ}\frac{{\left(ƻ-s\right)}^{\alpha -1}S_{1}(s,{℘}_{1}(\vartheta (s)),\vartheta_{1}(s-\sigma_{1}))}{\Gamma (\alpha )}ds,\frac{\rho_{2}{ƻ}^{\gamma -1}}{\Gamma (\gamma )}+\int_{0}^{ƻ}\frac{{\left(ƻ-s\right)}^{\alpha -1}S_{2}(s,{℘}_{2}(\vartheta (s)),\vartheta_{2}(s-\sigma_{2}))}{\Gamma (\alpha )}ds\right)$$ (22)

$$=\left(\frac{{\rho_{1}ƻ}^{\gamma -1}}{\Gamma (\gamma )}+\int_{0}^{{ƻ}_{1}}\frac{{\left(ƻ-s\right)}^{\alpha -1}S_{1}(s,{℘}_{1}(\vartheta (s)),\vartheta_{1}(s-\sigma_{1}))}{\Gamma (\alpha )}ds+(I_{{ƻ}_{1}}^{\alpha }S_{1}(s,{℘}_{1}(\vartheta (s)),\vartheta_{1}(s-\sigma_{1})))(ƻ),\frac{{\rho_{2}ƻ}^{\gamma -1}}{\Gamma (\gamma )}+\int_{0}^{{ƻ}_{1}}\frac{{\left(ƻ-s\right)}^{\alpha -1}S_{2}(s,{℘}_{2}(\vartheta (s)),\vartheta_{2}(s-\sigma_{2}))}{\Gamma (\alpha )}ds+(I_{{ƻ}_{1}}^{\alpha }S_{2}(s,{℘}_{2}(\vartheta (s)),\vartheta_{2}(s-\sigma_{2})))(ƻ)\right).$$

Let $\vartheta (ƻ),\tilde{\vartheta }(ƻ)\in C[{ƻ}_{1},{ƻ}_{2}]$ for some ${ƻ}_{1}<{ƻ}_{2}\leq \sigma_{1}$, we see that

$${\left\|G\vartheta (ƻ)-G\tilde{\vartheta }(ƻ)\right\|}_{C[{ƻ}_{1},{ƻ}_{2}]}$$

$$={\left\|\frac{{ƻ}^{\gamma -1}(\rho_{1}-\rho_{2})}{\Gamma (\gamma )}(I_{{ƻ}_{1}}^{\alpha }S(ƻ,℘(\vartheta (ƻ)),\vartheta (ƻ-\theta ))-I_{{ƻ}_{1}}^{\alpha }S(ƻ,℘(\tilde{\vartheta }(ƻ)),\tilde{\vartheta }(ƻ-\theta )))\right\|}_{C[{ƻ}_{1},{ƻ}_{2}]}$$

$$\leq \frac{\left|\left(\rho_{1}-\rho_{2}\right)\right|}{\Gamma (\gamma )}\frac{{\Gamma (\gamma )({ƻ}_{2}-{ƻ}_{1})}^{\gamma +\alpha -1}}{\Gamma (\gamma +\alpha )}{\left\|S(ƻ,℘(\vartheta (ƻ)),\vartheta (ƻ-\theta ))-S(ƻ,℘(\tilde{\vartheta }(ƻ)),\tilde{\vartheta }(ƻ-\theta ))\right\|}_{C[{ƻ}_{1},{ƻ}_{2}]}$$

$$\leq \frac{L{\left|\rho_{1}-\rho_{2}|({ƻ}_{2}-{ƻ}_{1}\right)}^{\gamma +\alpha -1}}{\Gamma (\gamma +\alpha )}({\left\|\vartheta (ƻ)-\tilde{\vartheta }(ƻ)\right\|}_{C[{ƻ}_{1},{ƻ}_{2}]}+{\left\|\vartheta (ƻ-\theta )-\tilde{\vartheta }(ƻ-\theta )\right\|}_{C[{ƻ}_{1},{ƻ}_{2}]})$$

$$=\frac{L{\left|\rho_{1}-\rho_{2}|({ƻ}_{2}-{ƻ}_{1}\right)}^{\gamma +\alpha -1}}{\Gamma (\gamma +\alpha )}({\left\|\vartheta (ƻ)-\tilde{\vartheta }(ƻ)\right\|}_{C[{ƻ}_{1},{ƻ}_{2}]}+{\left\|\varphi (ƻ-\theta )-\varphi (ƻ-\theta )\right\|}_{C[{ƻ}_{1},{ƻ}_{2}]})$$

$$=\frac{L{\left|\rho_{1}-\rho_{2}|({ƻ}_{2}-{ƻ}_{1}\right)}^{\gamma +\alpha -1}}{\Gamma (\gamma +\alpha )}{\left\|\vartheta (ƻ)-\tilde{\vartheta }(ƻ)\right\|}_{C[{ƻ}_{1},{ƻ}_{2}]}$$

$$=\mu_{2}{\left\|\vartheta (ƻ)-\tilde{\vartheta }(ƻ)\right\|}_{C[{ƻ}_{1},{ƻ}_{2}]},$$ (23)

where $\mu_{2}=\frac{L{\left|\rho_{1}-\rho_{2}|({ƻ}_{2}-{ƻ}_{1}\right)}^{\gamma +\alpha -1}}{\Gamma (\gamma +\alpha )}<1$. It results that *G* is a contraction on [ƻ_1_, ƻ_2_]. Consequently, ∃!-Sol *ϑ*_1_(ƻ) for ƻ∈[ƻ_1_, ƻ_2_]. According to Lemma 6, it is evident that $\vartheta_{0}({ƻ}_{1})=\vartheta_{1}({ƻ}_{1})$. Thus,

$$\vartheta (ƻ)=\left\{ \begin{array}{l} \vartheta_{0}(ƻ),0<ƻ\leq {ƻ}_{1},\\ \vartheta_{1}(ƻ),{ƻ}_{1}<ƻ\leq {ƻ}_{2}. \end{array}\right.$$ (24)

According to Lemma 7; $\vartheta (ƻ)\in C_{1-\gamma }[0,{ƻ}_{2}]$. Additionally, *ϑ*(ƻ) represents the only solution to (1) and (2) within [0,ƻ_2_]. In the case where ƻ_2_≠*σ*_1_, we repeat the necessary steps. Let’s assume that *N*−2 additional steps are required. Following that, we determine a! -Sol *ϑ*_*k*_(ƻ) for ƻ within $\left[{ƻ}_{k},{ƻ}_{k+1}\right]$, where *k* takes on values *k* = 2,3,⋯,*N*. Here, we have $0={ƻ}_{0}<{ƻ}_{1}<\cdots <{ƻ}_{N}=\sigma_{1}$ satisfying the condition that

$$\mu_{k+1}=\frac{L|\rho_{1}-\rho_{2}|}{\Gamma (\gamma +\alpha )}{\left({ƻ}_{k+1}-{ƻ}_{k}\right)}^{\gamma +\alpha -1}<1.$$ (25)

We possess a distinctive solution $\vartheta (ƻ)\in C_{1-\gamma }[0,\sigma_{1}]$ ensuring its uniqueness as

$$\vartheta (ƻ)=\left\{ \begin{array}{l} \vartheta_{0}(ƻ),0<ƻ<{ƻ}_{1},\\ \vartheta_{1}(ƻ),{ƻ}_{1}<ƻ\leq {ƻ}_{2},\\ \vdots \\ \vartheta_{N}(ƻ),{ƻ}_{N-1}<ƻ<\sigma_{1}. \end{array}\right.$$ (26)

Since $\vartheta (ƻ)\in C_{1-\gamma }[0,\tau ]$ is the! -Sol, so it fulfills (9) and

$$\vartheta (ƻ)=\left(\frac{\rho_{1}{ƻ}^{\gamma -1}}{\Gamma (\gamma )}+(I_{0^{+}}^{\alpha }S_{1}(s,{℘}_{1}(\vartheta (s)),\vartheta_{1}(s-\sigma_{1})))(ƻ),\frac{\rho_{2}{ƻ}^{\gamma -1}}{\Gamma (\gamma )}+(I_{0^{+}}^{\alpha }S_{2}(s,{℘}_{2}(\vartheta (s)),\vartheta_{2}(s-\sigma_{2})))(ƻ).\right)$$ (27)

Applying ${\;}^{L}D_{0^{+}}^{\gamma }$ to both sides results in the following:

$${\;}^{L}D_{0^{+}}^{\gamma }\vartheta (ƻ)=\left({\;}^{L}D_{0^{+}}^{\gamma }\frac{\rho_{1}{ƻ}^{\gamma -1}}{\Gamma (\gamma )}+{\;}^{L}D_{0^{+}}^{\gamma }(I_{0^{+}}^{\alpha }S_{1}(s,{℘}_{1}(\vartheta (s)),\vartheta_{1}(s-\sigma_{1})))(ƻ),{\;}^{L}D_{0^{+}}^{\gamma }\frac{\rho_{2}{ƻ}^{\gamma -1}}{\Gamma (\gamma )}+{\;}^{L}D_{0^{+}}^{\gamma }(I_{0^{+}}^{\alpha }S_{2}(s,{℘}_{2}(\vartheta (s)),\vartheta_{2}(s-\sigma_{2})))(ƻ)\right)$$

$$=\left(({\;}^{L}D_{0^{+}}^{\gamma -\alpha }S_{1}(s,{℘}_{1}(\vartheta (s)),\vartheta_{1}(s-\sigma_{1})))(ƻ),({\;}^{L}D_{0^{+}}^{\gamma -\alpha }S_{2}(s,{℘}_{2}(\vartheta (s)),\vartheta_{2}(s-\sigma_{2})))(ƻ)\right)$$

$$=(({\;}^{L}D_{0^{+}}^{\beta (1-\alpha )}S_{1}(s,{℘}_{1}(\vartheta (s)),\vartheta_{1}(s-\sigma_{1})))(ƻ),({\;}^{L}D_{0^{+}}^{\beta (1-\alpha )}S_{2}(s,{℘}_{2}(\vartheta (s)),\vartheta_{2}(s-\sigma_{2})))(ƻ)).$$ (28)

It is evident that the RHS belongs to *C*_1−*γ*_[0, *σ*_1_]. Therefore, ${\;}^{L}D_{0^{+}}^{\gamma }\vartheta (ƻ)\in C_{1-\gamma }[0,\sigma_{1}]$. Consequently, according to (9); $\vartheta (ƻ)\in C_{1-\gamma }^{\gamma }[0,\sigma_{1}]$ and this is the only solution in $\left[0,\sigma_{1}\right]$.

**Case 2.** Assuming that ƻ∈ (*σ*_1_,*σ*_2_] and $\vartheta (ƻ),\tilde{\vartheta }(ƻ)\in C_{1-\gamma }[\sigma_{1},\sigma_{2}]$, we can divide [0, *σ*_1_] into $\left[0,\sigma_{1}]\cup \cdots \cup [(k_{0}-1)\sigma_{1},k_{0}\sigma_{1}]\cup [k_{0}\sigma_{1},\sigma_{2}\right]$. Here, *k*_0_ is a natural number and fulfills $0\leq \sigma_{2}-k_{0}\sigma_{1}\leq \sigma_{2}$. In a previous case (Case 1), we have already proven (1) and (2) have a! -Sol on [0,*σ*_1_], denoted as $\vartheta_{0}^{*}(ƻ)$. Now, let’s assume that (1) and (2) also has a! -Sol on [*σ*_1_, *kσ*_1_], where 1≤*k*≤*k*_0_, denoted as $\vartheta_{k}^{*}(ƻ)$. Our goal is to axiomatize the existence of a! -Sol $\vartheta_{k+1}^{*}(ƻ)$ on $[k\sigma_{1},(k+1)\sigma_{1}].$ Anyhow, $\forall ƻ\in [k\sigma_{1},(k+1)\sigma_{1}]$ define

$$G\vartheta (ƻ)=(\frac{\rho_{1}{ƻ}^{\gamma -1}}{\Gamma (\gamma )}+\int_{0}^{ƻ}\frac{{\left(ƻ-s\right)}^{\alpha -1}S_{1}(s,{℘}_{1}(\vartheta (s)),\vartheta_{1}(s-\sigma_{1}))}{\Gamma (\alpha )}ds,\frac{\rho_{2}{ƻ}^{\gamma -1}}{\Gamma (\gamma )}+\int_{0}^{ƻ}\frac{{\left(ƻ-s\right)}^{\alpha -1}S_{2}(s,{℘}_{2}(\vartheta (s)),\vartheta_{2}(s-\sigma_{2}))}{\Gamma (\alpha )}ds).$$ (29)

Assume that G is a contraction for ƻ∈[*σ*_1_, *kσ*_1_] such that ${\left\|G\vartheta (ƻ)-G\tilde{\vartheta }(ƻ)\right\|}_{C[\sigma_{1},k\sigma_{1}]}\leq \mu_{k}{\left\|\vartheta (ƻ)-\tilde{\vartheta }(ƻ)\right\|}_{C[\sigma_{1},k\sigma_{1}]}$. Therefore, ∃!-Sol *φ*_*k*_(ƻ) for all ƻ∈[*σ*_1_, *kσ*_1_]. Now, consider $ƻ\in [k\sigma_{1},(k+1)\sigma_{1}]$. In this case, $\vartheta (ƻ-\sigma_{1})=\tilde{\vartheta }(ƻ-\sigma_{1})=\varphi_{k}(ƻ-\sigma_{1})$. Choose two values $c_{1},c_{2}$ such that $\sigma_{1}<c_{1}\leq c_{2}\leq k\sigma_{1}$. This entails that the metric space $C_{1-\gamma }[c_{1},c_{2}]$ is complete with

$$d(\vartheta (ƻ),\tilde{\vartheta }(ƻ))={\left\|\vartheta (ƻ)-\tilde{\vartheta }(ƻ)\right\|}_{C_{1-\gamma }[c_{1},c_{2}]}=\underset{ƻ\in [c_{1},c_{2}]}{\max}\left\|{ƻ}^{1-\gamma }(\vartheta (ƻ)-\tilde{\vartheta }(ƻ))\right\|.$$ (30)

Select ${ƻ}_{1}\in (k\sigma_{1},(k+1)\sigma_{1}]$ such that

$$\mu_{1k}=\frac{|\rho_{1}-\rho_{2}|L}{\Gamma (\gamma +\alpha )}{{ƻ}_{1}}^{\gamma +\alpha -1}<1.$$ (31)

Note that $\frac{\rho_{1}{ƻ}^{\gamma -1}}{\Gamma (\gamma )},\frac{\rho_{2}{ƻ}^{\gamma -1}}{\Gamma (\gamma )}\in C_{1-\gamma }[k\sigma_{1},{ƻ}_{1}]$, so by Lemma 5 due to $G\vartheta \in C_{1-\gamma }[k\sigma_{1},{ƻ}_{1}]$; *G* maps $C_{1-\gamma }[k\sigma_{1},{ƻ}_{1}]$ into itself. Now, $\forall \vartheta (ƻ),\tilde{\vartheta }(ƻ)\in C_{1-\gamma }[k\sigma_{1},{ƻ}_{1}]$, we have

$${\left\|G\vartheta (ƻ)-G\tilde{\vartheta }(ƻ)\right\|}_{C_{1-\gamma }[k\sigma_{1},{ƻ}_{1}]}\leq \frac{\left|\rho_{1}-\rho_{2}\right|}{\Gamma (\gamma )}{\left\|I_{0^{+}}^{\alpha }|S(ƻ,℘(\vartheta (ƻ)),\vartheta (ƻ-\sigma_{1}))-S(ƻ,℘(\tilde{\vartheta }(ƻ)),\tilde{\vartheta }(ƻ-\sigma_{1}))|\right\|}_{C_{1-\gamma }[k\sigma_{1},{ƻ}_{1}]}.$$ (32)

Note that

$${\left\|S(ƻ,℘(\vartheta (ƻ)),\vartheta (ƻ-\sigma_{1}))-S(ƻ,℘(\tilde{\vartheta }(ƻ)),\tilde{\vartheta }(ƻ-\sigma_{1}))\right\|}_{C_{1-\gamma }[k\sigma_{1},{ƻ}_{1}]}=\underset{ƻ\in [k\sigma_{1},{ƻ}_{1}]}{\max}\left\|{ƻ}^{1-\gamma }S(ƻ,℘(\vartheta (ƻ)),\vartheta (ƻ-\sigma_{1}))-S(ƻ,℘(\tilde{\vartheta }(ƻ)),\tilde{\vartheta }(ƻ-\sigma_{1}))\right\|.$$ (33)

Similarly, by doing the same procedure as in Case 1, we can find that

$${\left\|G\vartheta (ƻ)-G\tilde{\vartheta }(ƻ)\right\|}_{C_{1-\gamma }[k\sigma_{1},{ƻ}_{1}]}\leq \frac{\left|\rho_{1}-\rho_{2}\right|}{\Gamma (\gamma +\alpha )}{{ƻ}_{1}}^{\gamma +\alpha -1}L{\left\|\vartheta (ƻ)-\tilde{\vartheta }(ƻ)\right\|}_{C_{1-\gamma }[k\sigma_{1},{ƻ}_{1}]}$$

$$=\mu_{1k}{\left\|\vartheta (ƻ)-\tilde{\vartheta }(ƻ)\right\|}_{C_{1-\gamma }[k\sigma_{1},{ƻ}_{1}]},$$ (34)

where $\mu_{1k}=\frac{|\rho_{1}-\rho_{2}|L}{\Gamma (\gamma +\alpha )}{{ƻ}_{1}}^{\gamma +\alpha -1}<1$.

According to the CMT, there exists a single fixed point that serves as the solution $\vartheta_{0}(ƻ)\in C_{1-\gamma }[k\sigma_{1},{ƻ}_{1}]$ within $\left(k\sigma_{1},{ƻ}_{1}\right]$. In the case where ${ƻ}_{1}\neq (k+1)\sigma_{1}$, we will focus on ${[ƻ}_{1},(k+1)\sigma_{1}]$. It is important to note that

$$G\vartheta (ƻ)=(\frac{{\rho_{1}ƻ}^{\gamma -1}}{\Gamma (\gamma )}+\int_{0}^{{ƻ}_{1}}\frac{{\left(ƻ-s\right)}^{\alpha -1}S_{1}(s,{℘}_{1}(\vartheta (s)),\vartheta_{1}(s-\sigma_{1}))}{\Gamma (\alpha )}ds+(I_{{ƻ}_{1}}^{\alpha }S_{1}(s,{℘}_{1}(\vartheta (s)),\vartheta_{1}(s-\sigma_{1})))(ƻ),\frac{{\rho_{2}ƻ}^{\gamma -1}}{\Gamma (\gamma )}+\int_{0}^{{ƻ}_{1}}\frac{{\left(ƻ-s\right)}^{\alpha -1}S_{2}(s,{℘}_{2}(\vartheta (s)),\vartheta_{2}(s-\sigma_{2}))}{\Gamma (\alpha )}ds+(I_{{ƻ}_{1}}^{\alpha }S_{2}(s,{℘}_{2}(\vartheta (s)),\vartheta_{2}(s-\sigma_{2})))(ƻ)).$$ (35)

Let $\vartheta (ƻ),\tilde{\vartheta }(ƻ)\in C[{ƻ}_{1},{ƻ}_{2}]$ for some ${ƻ}_{1}<{ƻ}_{2}\leq (k+1)\sigma_{1}$, we can see that

$${\left\|G\vartheta (ƻ)-G\tilde{\vartheta }(ƻ)\right\|}_{C[{ƻ}_{1},{ƻ}_{2}]}$$

$$\leq |\frac{\left(\rho_{1}-\rho_{2}\right)}{\Gamma (\gamma )}|{\left\|I_{{{ƻ}_{1}}^{+}}^{\alpha }|S(ƻ,℘(\vartheta (ƻ)),\vartheta (ƻ-\theta ))-S(ƻ,℘(\tilde{\vartheta }(ƻ)),\tilde{\vartheta }(ƻ-\theta ))|\right\|}_{C[{ƻ}_{1},{ƻ}_{2}]}$$

$$\leq \frac{L{\left|\rho_{1}-\rho_{2}|({ƻ}_{2}-{ƻ}_{1}\right)}^{\gamma +\alpha -1}}{\Gamma (\gamma +\alpha )}({\left\|\vartheta (ƻ)-\tilde{\vartheta }(ƻ)\right\|}_{C[{ƻ}_{1},{ƻ}_{2}]}+{\left\|\vartheta (ƻ-\theta )-\tilde{\vartheta }(ƻ-\theta )\right\|}_{C[{ƻ}_{1},{ƻ}_{2}]})$$

$$=\frac{L{\left|\rho_{1}-\rho_{2}|({ƻ}_{2}-{ƻ}_{1}\right)}^{\gamma +\alpha -1}}{\Gamma (\gamma +\alpha )}({\left\|\vartheta (ƻ)-\tilde{\vartheta }(ƻ)\right\|}_{C[{ƻ}_{1},{ƻ}_{2}]}+{\left\|\varphi_{k}(ƻ-\theta )-\varphi_{k}(ƻ-\theta )\right\|}_{C[{ƻ}_{1},{ƻ}_{2}]})$$

$$=\mu_{2k}{\left\|\vartheta (ƻ)-\tilde{\vartheta }(ƻ)\right\|}_{C[{ƻ}_{1},{ƻ}_{2}]}.$$ (36)

such that $\mu_{2k}=\frac{L{\left|\rho_{1}-\rho_{2}|({ƻ}_{2}-{ƻ}_{1}\right)}^{\gamma +\alpha -1}}{\Gamma (\gamma +\alpha )}<1$, it results that *G* is a contraction on [ƻ_1_,ƻ_2_].

Consequently, ∃!-Sol *ϑ*_1_(ƻ) for ƻ∈[ƻ_1_,ƻ_2_]. According to Lemma 6, it can be observed that $\vartheta_{0}({ƻ}_{1})=\vartheta_{1}({ƻ}_{1})$. Therefore,

$$\vartheta (ƻ)=\left\{ \begin{array}{l} \vartheta_{0}(ƻ),0<ƻ\leq {ƻ}_{1},\\ \vartheta_{1}(ƻ),{ƻ}_{1}<ƻ\leq {ƻ}_{2}. \end{array}\right.$$ (37)

Using Lemma 7, $\vartheta (ƻ)\in C_{1-\gamma }[k\sigma_{1},{ƻ}_{2}]$. Therefore, *ϑ*(ƻ) is the only solution of (1) and (2) over $\left[k\sigma_{1},{ƻ}_{2}\right]$. If ${ƻ}_{2}\neq (k+1)\sigma_{1}$, we proceed with the steps as required. Let’s presume that we require *N*−2 additional steps. Following, we discover the distinct solution *ϑ*_*k*_(ƻ) for $ƻ\in [{ƻ}_{k},{ƻ}_{k+1}],k=2,3,\cdots ,N$, where $k\sigma_{1}={ƻ}_{0}<{ƻ}_{1}<\cdots <{ƻ}_{N}=(k+1)\sigma_{1}$ such that

$$\mu_{k+1}=\frac{L|\rho_{1}-\rho_{2}|}{\Gamma (\gamma +\alpha )}{\left({ƻ}_{k+1}-{ƻ}_{k}\right)}^{\gamma +\alpha -1}<1.$$ (38)

We possess the exclusive solution $\vartheta (ƻ)\in C_{1-\gamma }[k\sigma_{1},(k+1)\sigma_{1}]$ such that

$$\vartheta (ƻ)=\left\{ \begin{array}{l} \vartheta_{0}(ƻ),k\sigma_{1}<ƻ<{ƻ}_{1},\\ \vartheta_{1}(ƻ),{ƻ}_{1}<ƻ\leq {ƻ}_{2},\\ \vdots \\ \vartheta_{N}(ƻ),{ƻ}_{N-1}<ƻ<(k+1)\sigma_{1}. \end{array}\right.$$ (39)

Since $\vartheta (ƻ)\in C_{1-\gamma }[k\sigma_{1},(k+1)\sigma_{1}]$ is the! -Sol, so it fulfills (9) and

$$\vartheta (ƻ)=(\frac{\rho_{1}{ƻ}^{\gamma -1}}{\Gamma (\gamma )}+(I_{k\tau }^{\alpha }S_{1}(s,{℘}_{1}(\vartheta (s)),\vartheta_{1}(s-\sigma_{1})))(ƻ),\frac{\rho_{2}{ƻ}^{\gamma -1}}{\Gamma (\gamma )}+(I_{k\tau }^{\alpha }S_{2}(s,{℘}_{2}(\vartheta (s)),\vartheta_{2}(s-\sigma_{2})))(ƻ)).$$ (40)

Applying ${\;}^{L}D_{{k\sigma_{1}}^{+}}^{\gamma }$ to (40) gives

$${\;}^{L}D_{{k\tau }^{+}}^{\gamma }\vartheta (ƻ)=(({\;}^{L}D_{{k\sigma_{1}}^{+}}^{\beta (1-\alpha )}S_{1}(s,{℘}_{1}(\vartheta (s)),\vartheta_{1}(s-\sigma_{1})))(ƻ),({\;}^{L}D_{{k\sigma_{1}}^{+}}^{\beta (1-\alpha )}S_{2}(s,{℘}_{2}(\vartheta (s)),\vartheta_{2}(s-\sigma_{2})))(ƻ)).$$ (41)

It is observed that the RHS lies within the range of $C_{1-\gamma }[k\sigma_{1},(k+1)\sigma_{1}]$ which entails that ${\;}^{L}D_{{k\sigma_{1}}^{+}}^{\gamma }\vartheta (ƻ)\in C_{1-\gamma }[k\sigma_{1},(k+1)\sigma_{1}]$. Consequently, according to (9); $\vartheta (ƻ)\in C_{1-\gamma }^{\gamma }[k\sigma_{1},(k+1)\sigma_{1}]$ and this is the only solution for $\left[k\sigma_{1},(k+1)\sigma_{1}\right]$. By applying induction, a! -Sol exists for [*σ*_1_, *σ*_2_].

**Case 3.** When $ƻ\in (\sigma_{2},F]$ and $\vartheta (ƻ),\tilde{\vartheta }(ƻ)\in C_{1-\gamma }[\sigma_{2},F]$ we can divide [0,ℱ] into $\left[0,\sigma_{2}]\cup \cdots \cup [(k_{0}-1)\sigma_{2},k_{0}\sigma_{2}]\cup [k_{0}\sigma_{2},F\right]$, where *k*_0_∈ℕ and fulfills $0\leq F-k_{0}\sigma_{2}\leq F$. In Case 1, we have already proven that (1) and (2) have a! -Sol on [0, *σ*_2_], denoted as $\vartheta_{0}^{*}(ƻ)$. Now, let’s assume that (1) and (2) have a! -Sol on [*σ*_2_, *kσ*_2_], where 1≤*k*≤*k*_0_ say $\vartheta_{k}^{*}(ƻ)$. We aim to ensure the existence of a! -Sol $\vartheta_{k+1}^{*}(ƻ)$ on $[k\sigma_{2},(k+1)\sigma_{2}].$ Consider $ƻ\in [k\sigma_{2},(k+1)\sigma_{2}]$ and

$$G\vartheta (ƻ)=(\frac{\rho_{1}{ƻ}^{\gamma -1}}{\Gamma (\gamma )}+\int_{0}^{ƻ}\frac{{\left(ƻ-s\right)}^{\alpha -1}S_{1}(s,{℘}_{1}(\vartheta (s)v),\vartheta_{1}(s-\sigma_{1}))}{\Gamma (\alpha )}ds,\frac{\rho_{2}{ƻ}^{\gamma -1}}{\Gamma (\gamma )}+\int_{0}^{ƻ}\frac{{\left(ƻ-s\right)}^{\alpha -1}S_{2}(s,{℘}_{2}(\vartheta (s)),\vartheta_{2}(s-\sigma_{2}))}{\Gamma (\alpha )}ds).$$ (42)

Suppose *G* is a contraction for ƻ in [*σ*_2_, *kσ*_2_] such that ${\left\|G\vartheta (ƻ)-G\tilde{\vartheta }(ƻ)\right\|}_{C[\sigma_{2},k\sigma_{2}]}\leq \mu_{k}{\left\|\vartheta (ƻ)-\tilde{\vartheta }(ƻ)\right\|}_{C[\sigma_{2},k\sigma_{2}]}$. This entails that ∃!-Sol $\varphi_{k}(ƻ)=(\varphi_{1k}(ƻ),\varphi_{2k}(ƻ))$ for all $ƻ\in [\sigma_{2},k\sigma_{2}]$. Consider $ƻ\in [k\sigma_{2},(k+1)\sigma_{2}]$, so, $\vartheta (ƻ-\sigma_{2})=\tilde{\vartheta }(ƻ-\sigma_{2})=\varphi_{k}(ƻ-\sigma_{2})=(\varphi_{1k}(ƻ-\sigma_{2}),\varphi_{2k}(ƻ-\sigma_{2}))$. Let $c_{1}$ and $c_{2}$ be chosen such that $\sigma <c_{1}\leq c_{2}\leq k\sigma_{2}$. Then, $C_{1-\gamma }[c_{1},c_{2}]$ equipped

$$d(\vartheta (ƻ),\tilde{\vartheta }(ƻ))={\left\|\vartheta (ƻ)-\tilde{\vartheta }(ƻ)\right\|}_{C_{1-\gamma }[c_{1},c_{2}]}=\underset{ƻ\in [c_{1},c_{2}]}{\max}\left\|{ƻ}^{1-\gamma }(\vartheta (ƻ)-\tilde{\vartheta }(ƻ))\right\|.$$ (43)

Select ${ƻ}_{1}\in (k\sigma_{2},(k+1)\sigma_{2}]$ and due to $\frac{\rho_{1}{ƻ}^{\gamma -1}}{\Gamma (\gamma )},\frac{\rho_{2}{ƻ}^{\gamma -1}}{\Gamma (\gamma )}\in C_{1-\gamma }[k\sigma_{2},{ƻ}_{1}]$, so leveraging Lemma 5 due to $G\vartheta \in C_{1-\gamma }[k\sigma_{2},{ƻ}_{1}]$; G maps $C_{1-\gamma }[k\sigma_{2},{ƻ}_{1}]$ into itself. Therefore, $\forall \vartheta (ƻ),\tilde{\vartheta }(ƻ)\in C_{1-\gamma }[k\sigma_{2},{ƻ}_{1}]$, we have

$${\left\|G\vartheta (ƻ)-G\tilde{\vartheta }(ƻ)\right\|}_{C_{1-\gamma }[k\sigma_{2},{ƻ}_{1}]}={\left\|I_{0^{+}}^{\alpha }S(ƻ,℘(\vartheta (ƻ)),\vartheta (ƻ-\theta ))-I_{0^{+}}^{\alpha }S(ƻ,℘(\tilde{\vartheta }(ƻ)),\tilde{\vartheta }(ƻ-\theta ))\right\|}_{C_{1-\gamma }[k\sigma_{2},{ƻ}_{1}]}$$

$$\leq {\left\|I_{0^{+}}^{\alpha }|S(ƻ,℘(\vartheta (ƻ)),\vartheta (ƻ-\theta ))-S(ƻ,℘(\tilde{\vartheta }(ƻ)),\tilde{\vartheta }(ƻ-\theta ))|\right\|}_{C_{1-\gamma }[k\sigma_{2},{ƻ}_{1}]}.$$ (44)

Note that

$${\left\|S(ƻ,℘(\vartheta (ƻ)),\vartheta (ƻ-\theta ))-S(ƻ,℘(\tilde{\vartheta }(ƻ)),\tilde{\vartheta }(ƻ-\theta ))\right\|}_{C_{1-\gamma }[k\sigma_{2},{ƻ}_{1}]}=\underset{ƻ\in [k\sigma_{2},{ƻ}_{1}]}{\max}\left\|{ƻ}^{1-\gamma }S(ƻ,℘(\vartheta (ƻ)),\vartheta (ƻ-\theta ))-S(ƻ,℘(\tilde{\vartheta }(ƻ)),\tilde{\vartheta }(ƻ-\theta ))\right\|.$$ (45)

Similarly, by applying the same procedure in Cases 1 and 2, one finds

$${\left\|G\vartheta (ƻ)-G\tilde{\vartheta }(ƻ)\right\|}_{C_{1-\gamma }[k\sigma_{2},{ƻ}_{1}]}\leq \frac{\left|\rho_{1}-\rho_{2}\right|}{\Gamma (\gamma +\alpha )}{{ƻ}_{1}}^{\gamma +\alpha -1}L{\left\|\vartheta (ƻ)-\tilde{\vartheta }(ƻ)\right\|}_{C_{1-\gamma }[k\sigma_{2},{ƻ}_{1}]}=\mu_{1k}{\left\|\vartheta (ƻ)-\tilde{\vartheta }(ƻ)\right\|}_{C_{1-\gamma }[k\sigma_{2},{ƻ}_{1}]},$$ (46)

where $\mu_{1k}=\frac{L|\rho_{1}-\rho_{2}|}{\Gamma (\gamma +\alpha )}{{ƻ}_{1}}^{\gamma +\alpha -1}<1$.

According to the CMT, there exists a single fixed point that serves as the solution $\vartheta_{0}(ƻ)\in C_{1-\gamma }[k\sigma_{2},{ƻ}_{1}]$ within (*kσ*, ƻ_1_]. In the case where ${ƻ}_{1}\neq (k+1)\sigma_{2}$ we examine ${[ƻ}_{1},(k+1)\sigma_{2}]$. It is important to note that

$$G\vartheta (ƻ)=(\frac{{\rho_{1}ƻ}^{\gamma -1}}{\Gamma (\gamma )}+\int_{0}^{{ƻ}_{1}}\frac{{\left(ƻ-s\right)}^{\alpha -1}S_{1}(s,{℘}_{1}(\vartheta (s)),\vartheta_{1}(s-\sigma_{1}))}{\Gamma (\alpha )}ds+(I_{{ƻ}_{1}}^{\alpha }S_{1}(s,{℘}_{1}(\vartheta (s)),\vartheta_{1}(s-\sigma_{1})))(ƻ),\frac{{\rho_{2}ƻ}^{\gamma -1}}{\Gamma (\gamma )}+\int_{0}^{{ƻ}_{1}}\frac{{\left(ƻ-s\right)}^{\alpha -1}S_{2}(s,{℘}_{2}(\vartheta (s)),\vartheta_{2}(s-\sigma_{2}))}{\Gamma (\alpha )}ds+(I_{{ƻ}_{1}}^{\alpha }S_{2}(s,{℘}_{2}(\vartheta (s)),\vartheta_{2}(s-\sigma_{2})))(ƻ)).$$ (47)

Let $\vartheta (ƻ),\tilde{\vartheta }(ƻ)\in C[{ƻ}_{1},{ƻ}_{2}]$ for some ${ƻ}_{1}<{ƻ}_{2}\leq (k+1)\sigma_{2}$, we see that

$${\left\|G\vartheta (ƻ)-G\tilde{\vartheta }(ƻ)\right\|}_{C[{ƻ}_{1},{ƻ}_{2}]}$$

$$\leq \frac{\left|\rho_{1}-\rho_{2}\right|}{\Gamma (\gamma )}\frac{\Gamma (\gamma ){\left({ƻ}_{2}-{ƻ}_{1}\right)}^{\gamma +\alpha -1}}{\Gamma (\alpha +\gamma )}{\left\|S(ƻ,℘(\vartheta (ƻ)),\vartheta (ƻ-\theta ))-S(ƻ,℘(\tilde{\vartheta }(ƻ)),\tilde{\vartheta }(ƻ-\theta ))\right\|}_{C[{ƻ}_{1},{ƻ}_{2}]}$$

$$\leq \frac{L|\rho_{1}-\rho_{2}|{\left({ƻ}_{2}-{ƻ}_{1}\right)}^{\gamma +\alpha -1}}{\Gamma (\alpha +\gamma )}({\left\|\vartheta (ƻ)-\tilde{\vartheta }(ƻ)\right\|}_{C[{ƻ}_{1},{ƻ}_{2})}+{\left\|\vartheta (ƻ-\theta )-\tilde{\vartheta }(ƻ-\theta )\right\|}_{C[{ƻ}_{1},{ƻ}_{2}]})$$

$$=\frac{L|\rho_{1}-\rho_{2}|{\left({ƻ}_{2}-{ƻ}_{1}\right)}^{\gamma +\alpha -1}}{\Gamma (\alpha +\gamma )}({\left\|\vartheta (ƻ)-\tilde{\vartheta }(ƻ)\right\|}_{C[{ƻ}_{1},{ƻ}_{2}]}+{\left\|\varphi_{k}(ƻ-\theta )-\varphi_{k}(ƻ-\theta )\right\|}_{C[{ƻ}_{1},{ƻ}_{2}]})$$

$$=\mu_{2k}{\left\|\vartheta (ƻ)-\tilde{\vartheta }(ƻ)\right\|}_{C[{ƻ}_{1},{ƻ}_{2}]}.$$ (48)

where $\mu_{2k}=\frac{L|\rho_{1}-\rho_{2}|{\left({ƻ}_{2}-{ƻ}_{1}\right)}^{\gamma +\alpha -1}}{\Gamma (\alpha +\gamma )}<1$. It results that *G* is a contraction over [ƻ_1_,ƻ_2_], and there exists a single solution *ϑ*_1_(ƻ) for ƻ∈[ƻ_1_,ƻ_2_]. As per Lemma 6, it can be observed that *ϑ*_0_(ƻ_1_) = *ϑ*_1_(ƻ_1_). Consequently, the function *ϑ*_1_(ƻ) can be expressed as

$$\vartheta (ƻ)=\left\{ \begin{array}{l} \vartheta_{0}(ƻ),0<ƻ\leq {ƻ}_{1},\\ \vartheta_{1}(ƻ),{ƻ}_{1}<ƻ\leq {ƻ}_{2}. \end{array}\right.$$ (49)

Furthermore, according to Lemma 7; $\vartheta (ƻ)\in C_{1-\gamma }[k\sigma_{2},{ƻ}_{2}]$. Therefore, *ϑ*(ƻ) represents the! -Sol to (1) and (2) within [*kσ*, ƻ_2_]. If ${ƻ}_{2}\neq (k+1)\sigma_{2}$ the aforementioned steps can be repeated as necessary. Assuming that *N*−2 additional steps are required; the! -Sol *ϑ*_*k*_(ƻ) can be determined for $ƻ\in [{ƻ}_{k},{ƻ}_{k+1}]$, where *k* = 2,3,…,*N*. Here, $k\sigma_{2}={ƻ}_{0}<{ƻ}_{1}<\cdots <{ƻ}_{N}=(k+1)\sigma_{2}$ ensuring the resulting conditions are satisfied

$$\mu_{k+1}=\frac{L|\rho_{1}-\rho_{2}|}{\Gamma (\gamma +\alpha )}{\left({ƻ}_{k+1}-{ƻ}_{k}\right)}^{\gamma +\alpha -1}<1.$$ (50)

We possess a! -Sol $\vartheta (ƻ)\in C_{1-\gamma }[k\sigma_{2},(k+1)\sigma_{2}]$ which can be defined as

$$\vartheta (ƻ)=\left\{ \begin{array}{l} \vartheta_{0}(ƻ),k\sigma_{2}<ƻ<{ƻ}_{1},\\ \vartheta_{1}(ƻ),{ƻ}_{1}<ƻ\leq {ƻ}_{2},\\ \vdots \\ \vartheta_{N}(ƻ),{ƻ}_{N-1}<ƻ<(k+1)\sigma_{2}. \end{array}\right.$$ (51)

Since $\vartheta (ƻ)\in C_{1-\gamma }[k\sigma_{2},(k+1)\sigma_{2}]$ is the! -Sol, it fulfills (9) and

$$\vartheta (ƻ)=(\frac{\rho_{1}{ƻ}^{\gamma -1}}{\Gamma (\gamma )}+(I_{k\sigma_{2}}^{\alpha }S_{1}(s,{℘}_{1}(\vartheta (s)),\vartheta_{1}(s-\sigma_{1})))(ƻ),\frac{\rho_{2}{ƻ}^{\gamma -1}}{\Gamma (\gamma )}+(I_{k\sigma_{2}}^{\alpha }S_{2}(s,{℘}_{2}(\vartheta (s)),\vartheta_{2}(s-\sigma_{2})))(ƻ)).$$ (52)

Applying ${\;}^{L}D_{{k\sigma_{2}}^{+}}^{\gamma }$ to both sides of (52) gives

$${\;}^{L}D_{{k\sigma }^{+}}^{\gamma }\vartheta (ƻ)=(({\;}^{L}D_{{k\sigma_{2}}^{+}}^{\beta (1-\alpha )}S_{1}(s,{℘}_{1}(\vartheta (s)),\vartheta_{1}(s-\sigma_{1})))(ƻ),({\;}^{L}D_{{k\sigma_{2}}^{+}}^{\beta (1-\alpha )}S_{2}(s,{℘}_{2}(\vartheta (s)),\vartheta_{2}(s-\sigma_{2})))(ƻ)).$$ (53)

We can see that the RHS is in *C*_1−*γ*_. Consequently, ${\;}^{L}D_{{k\sigma_{2}}^{+}}^{\gamma }\vartheta (ƻ)\in C_{1-\gamma }[k\sigma_{2},(k+1)\sigma_{2}]$, so, based on (9); $\vartheta (ƻ)\in C_{1-\gamma }^{\gamma }[k\sigma_{2},(k+1)\sigma_{2}]]\;is\;the\;only\;solution\;for\;[k\sigma_{2},(k+1)\sigma_{2}]$. Applying induction, we can infer that a!-Sol exists for [*σ*_2_, ℱ ].

Finally, considering Cases (1–3), one can conclude that a! -Sol exists for (1) and (2) within $C_{1-\gamma }^{\gamma }(J)$.

## 4 The SGLA: Assembly and outcomes

This section introduces the SGLA numerical scheme for solving (1) and (2). This approach is a type of weighted residual numerical technique that uses a finite set of basis polynomials as weighting functions. Based on our proposed algorithm, we employ orthogonal spline local polynomial functions as the weighting functions. Indeed, we prove theorems regarding the convergence and error estimation of the SGLA.

Broadly, the SGLA is a numerical technique used to solve various classes of differential/integral problems including several types by approximating the solution by leveraging a finite collection of orthogonal basis functions. In implementing the Galerkin scheme, the orthogonal basis functions are selected to satisfy the initial or boundary constraints. The effectiveness of the Galerkin approach relies on several prerequisites as follows:

- The basis functions must be orthogonal, facilitating straightforward computation of solution coefficients.
- The selected basis functions should have regular behavior and adequate smoothness to ensure precise approximation.
- The Galerkin numerical scheme will provide accurate results if the problem has a! -Sol that depends continuously on the data and problem constraints. Failure to meet these constraints may result in imprecise or erroneous outcomes from the Galerkin approach.

Initially, we define the OSLPs as basis functions for representing functions within a predefined interval. Following that, we use this concept to define the essential functions in (1) in terms of OSLPs. Next, we calculate the residual required in the Galerkin numerical process. Leveraging the orthogonality of the residual with the OSLPs, the sets of FDDSs can be simplified into an algebraic one. Finally, this system can be solved by leveraging MATHEMATICA 11 to obtain the needed approximation.

**Definition 5.** [34] The OSLP of multiplicity ϐ is

$$\phi_{ϐ}(ƻ)=\sum_{ɷ=0}^{ϐ}‍(-1{)}^{ϐ+ɷ}\frac{\Gamma (\hbar +ɷ+1)}{\Gamma (\hbar -ɷ+1)(\Gamma (ɷ+1){)}^{2}}{ƻ}^{ɷ},$$ (54)

where $ɷ=0,1,\cdots ,ϐ,ƻ\in [0,1],\phi_{ϐ}(0)=(-1{)}^{ϐ}$, and *ϕ*_ϐ_(1) = 1.

The condition of orthogonality is defined as

$$\int_{0}^{1}‍\phi_{ϐ}(ƻ)\phi_{ɷ}(ƻ)dƻ=\left\{ \begin{array}{l} \frac{1}{2ϐ+1},ϐ=ɷ,\\ 0,ϐ\neq ɷ. \end{array}\right.$$ (55)

This entails that ∀Δ∈C([0,1]→R) may characterized in OSLPs with ϐ = 0,1,⋯,ℏ as

$$\left\{ \begin{array}{l} \Delta (ƻ)\approx \Delta_{\hbar }(ƻ)=(\Delta_{1}(ƻ),\Delta_{2}(ƻ))=\left(\sum_{ϐ=0}^{\hbar }‍a_{ϐ}\phi_{ϐ}(ƻ),\sum_{ϐ=0}^{\hbar }‍b_{ϐ}\phi_{ϐ}(ƻ)\right),\\ a_{ϐ}=\langle \Delta_{1}(ƻ),\phi_{ϐ}(ƻ)\rangle =\int_{0}^{1}‍(2ϐ+1)\Delta_{1}(ƻ)\phi_{ϐ}(ƻ)dƻ,\\ b_{ϐ}=\langle \Delta_{2}(ƻ),\phi_{ϐ}(ƻ)\rangle =\int_{0}^{1}‍(2ϐ+1)\Delta_{2}(ƻ)\phi_{ϐ}(ƻ)dƻ. \end{array}\right.$$ (56)

Now, interpolating OSLP for *ϑ*(ƻ) in (1), we get

$$\vartheta (ƻ)\approx \vartheta_{\hbar }(ƻ)=\left(\sum_{ϐ=0}^{\hbar }‍a_{ϐ}\phi_{ϐ}(ƻ),\sum_{ϐ=0}^{\hbar }‍b_{ϐ}\phi_{ϐ}(ƻ)\right),$$ (57)

where $a_{ϐ}$ and $b_{ϐ},$ ϐ = 0,1,⋯,ℏ are the unknown parameters.

Again, ${\;}^{H}D_{0^{+}}^{\alpha ,\beta }\vartheta (ƻ),℘(\vartheta (ƻ))$, and S(ƻ,℘(ϑ(ƻ)),ϑ(ƻ−θ)) can be approximated with

$${\;}^{H}D_{0^{+}}^{\alpha ,\beta }\vartheta (ƻ)\approx {\;}^{H}D_{0^{+}}^{\alpha ,\beta }\vartheta_{\hbar }(ƻ)=(\sum_{ϐ=0}^{\hbar }‍a_{ϐ}{\;}^{H}D_{0^{+}}^{\alpha ,\beta }\phi_{ϐ}(ƻ),\sum_{ϐ=0}^{\hbar }‍b_{ϐ}{\;}^{H}D_{0^{+}}^{\alpha ,\beta }\phi_{ϐ}(ƻ)).$$ (58)

$$℘(\vartheta (ƻ))\approx {℘}_{\hbar }(\vartheta_{\hbar }(ƻ))=({℘}_{1\hbar }(\vartheta_{\hbar }(ƻ)),{℘}_{2\hbar }(\vartheta_{\hbar }(ƻ))).$$ (59)

$$S(ƻ,℘(\vartheta (ƻ)),\vartheta (ƻ-\theta ))\approx S_{\hbar }(ƻ,{℘}_{\hbar }(\vartheta_{\hbar }(ƻ)),\vartheta_{\hbar }(ƻ-\theta ))=(S_{1}(ƻ,{℘}_{1\hbar }(\vartheta_{\hbar }(ƻ)),\sum_{ϐ=0}^{\hbar }‍a_{ϐ}\phi_{ϐ}(ƻ-\sigma_{1})),S_{2}(ƻ,{℘}_{2\hbar }(\vartheta_{\hbar }(ƻ)),\sum_{ϐ=0}^{\hbar }‍b_{ϐ}\phi_{ϐ}(ƻ-\sigma_{2}))).$$ (60)

Swapping out (57), (58), (59) and (60) into (1) gives

$${\;}^{H}D_{0^{+}}^{\alpha ,\beta }\vartheta_{\hbar }(ƻ)=S_{\hbar }(ƻ,{℘}_{\hbar }(\vartheta_{\hbar }(ƻ)),\vartheta_{\hbar }(ƻ-\theta )).$$ (61)

To execute the SGLA, simulate the residual, *R*, out of (61) as

$$R(ƻ)=(R_{1}(ƻ)-R_{2}(ƻ))={\;}^{H}D_{0^{+}}^{\alpha ,\beta }\vartheta_{\hbar }(ƻ)-S_{\hbar }(ƻ,{℘}_{\hbar }(\vartheta_{\hbar }(ƻ)),\vartheta_{\hbar }(ƻ-\theta )).$$ (62)

When *R*(ƻ) = (0,0), the result solves (1) and fulfills the presumed constraints, Therefore, in our research, we intend to optimize *R* to be zero in light of the selected OSLP that is chosen as the basis to axiomatize *ϑ*_ℏ_(ƻ).

To fix the unknowable parameters $a_{0},a_{1},\cdots ,a_{\hbar },b_{0},b_{1},\cdots ,b_{\hbar }$ in (57), we select weight functions as the granted basis and integrate constraint controls concerning *R* = 0. By leveraging the orthogonality of *R* at (ℏ+1) mapping $\phi_{0}(ƻ),\phi_{1}(ƻ),\cdots ,\phi_{\hbar }(ƻ)$ as

$$\left\{ \begin{array}{l} \int_{0}^{1}‍R_{1}(ƻ)\phi_{ϐ}(ƻ)dƻ=0,ϐ=0,1,\cdots ,\hbar -1,\\ \sum_{ϐ=0}^{\hbar }‍a_{ϐ}\phi_{ϐ}(0)=\phi_{1}(0). \end{array}\right.$$ (63)

$$\left\{ \begin{array}{l} \int_{0}^{1}‍R_{2}(ƻ)\phi_{ϐ}(ƻ)dƻ=0,ϐ=0,1,\cdots ,\hbar -1,\\ \sum_{ϐ=0}^{\hbar }‍b_{ϐ}\phi_{ϐ}(0)=\phi_{2}(0). \end{array}\right.$$ (64)

So, one has a set of 2(ℏ+1) algebraic equations including 2(ℏ+1) unknown $a_{0},a_{1},\cdots ,a_{\hbar },b_{0},b_{1},\cdots ,b_{\hbar }$. After substituting the estimated $a_{0},a_{1},\cdots ,a_{\hbar },b_{0},b_{1},\cdots ,b_{\hbar }$ parameters in (57) we get the approximated solution for (1).

Next, similar to symbolization in Theorem 2; $\vartheta_{\hbar }(ƻ)=(\vartheta_{1\hbar }(ƻ),\vartheta_{2\hbar }(ƻ))=(\sum_{ϐ=0}^{\hbar }‍a_{ϐ}\phi_{ϐ}(ƻ),\sum_{ϐ=0}^{\hbar }‍b_{ϐ}\phi_{ϐ}(ƻ)),\vartheta_{ҟ}(ƻ)=(\vartheta_{1ҟ}(ƻ),\vartheta_{2ҟ}(ƻ))=(\sum_{ϐ=0}^{ҟ}‍a_{ϐ}\phi_{ϐ}(ƻ),\sum_{ϐ=0}^{ҟ}‍b_{ϐ}\phi_{ϐ}(ƻ))$, and $\vartheta (ƻ)=(\vartheta_{1}(ƻ),\vartheta_{2}(ƻ))$.

**Theorem 3.** Let $\left(\sum_{ϐ=0}^{\infty }‍a_{ϐ}\phi_{ϐ}(ƻ),\sum_{ϐ=0}^{\infty }‍b_{ϐ}\phi_{ϐ}(ƻ)\right)$ be the Legendre interpolation of *ϑ*(ƻ) with $\vartheta_{ϐ}(ƻ)\in L^{2}[0,1]$. Then $\vartheta_{\hbar }(ƻ)\to \vartheta (ƻ)$ as ℏ→∞.

**Proof.** Set *ϑ*_ҟ_(ƻ) be a partial sum in $\left(\sum_{ϐ=0}^{\infty }‍a_{ϐ}\phi_{ϐ}(ƻ),\sum_{ϐ=0}^{\infty }‍b_{ϐ}\phi_{ϐ}(ƻ)\right)$. Then at ℏ>ҟ, it is straightforward to verify that

$${\left\|\vartheta_{\hbar }(ƻ)-\vartheta_{ҟ}(ƻ)\right\|}^{2}={\left\|\left[\begin{array}{c} \vartheta_{1\hbar }(ƻ)\\ \vartheta_{2\hbar }(ƻ) \end{array}]-[\begin{array}{c} \vartheta_{1ҟ}(ƻ)\\ \vartheta_{2ҟ}(ƻ) \end{array}\right]\right\|}^{2}={\left\|\left[\begin{array}{c} \sum_{ϐ=ҟ+1}^{\hbar }{a_{ϐ}\varphi }_{ϐ}(ƻ)\\ \sum_{ϐ=ҟ+1}^{\hbar }b_{ϐ}\varphi_{ϐ}(ƻ) \end{array}\right]\right\|}^{2}<[\begin{array}{c} \sum_{ϐ=ҟ+1}^{\hbar }{\left|a_{ϐ}\right|}^{2}\\ \sum_{ϐ=ҟ+1}^{\hbar }{\left|b_{ϐ}\right|}^{2} \end{array}].$$ (65)

Utilizing the inequality of Bessel’s, one finds $\sum_{ϐ=ҟ+1}^{\hbar }{\left|a_{ϐ}\right|}^{2}\leq \sum_{ϐ=ҟ+1}^{\infty }{\left|a_{ϐ}\right|}^{2}\leq {\left\|\vartheta \right\|}^{2}<\infty$ and $\sum_{ϐ=ҟ+1}^{\hbar }{\left|b_{ϐ}\right|}^{2}\leq \sum_{ϐ=ҟ+1}^{\infty }{\left|b_{ϐ}\right|}^{2}\leq {\left\|\vartheta \right\|}^{2}<\infty$. So, ${\left\|\vartheta_{\hbar }(ƻ)-\vartheta_{ҟ}(ƻ)\right\|}^{2}\to 0$ as ℏ,ҟ→∞. Thus, the sequence *ϑ*_ℏ_(ƻ) is Cauchy and converges to $S(ƻ)\in L^{2}(\left[0,1\right)]$. Anyhow,

$$\langle \left[\begin{array}{c} S_{1}(ƻ)\\ S_{2}(ƻ) \end{array}]-[\begin{array}{c} \vartheta_{1}(ƻ)\\ \vartheta_{2}(ƻ) \end{array}],[\begin{array}{c} \varphi_{ϐ}(ƻ)\\ \varphi_{ϐ}(ƻ) \end{array}\right]\rangle =\langle \left[\begin{array}{c} S_{1}(ƻ)\\ S_{2}(ƻ) \end{array}],[\begin{array}{c} \varphi_{ϐ}(ƻ)\\ \varphi_{ϐ}(ƻ) \end{array}\right]\rangle -\langle \left[\begin{array}{c} \vartheta_{1}(ƻ)\\ \vartheta_{2}(ƻ) \end{array}],[\begin{array}{c} \varphi_{ϐ}(ƻ)\\ \varphi_{ϐ}(ƻ) \end{array}\right]\rangle$$

$$=\underset{\hbar \to \infty }{\lim}\langle \left[\begin{array}{c} \vartheta_{1\hbar }(ƻ)\\ \vartheta_{2\hbar }(ƻ) \end{array}],[\begin{array}{c} \varphi_{ϐ}(ƻ)\\ \varphi_{ϐ}(ƻ) \end{array}\right]\rangle -(a_{ϐ},b_{ϐ})$$

$$=(a_{ϐ},b_{ϐ})-(a_{ϐ},b_{ϐ})$$

=(0,0). (66)

Hence, $\left(S_{1}(ƻ),S_{2}(ƻ)\right)$ and $\left(\vartheta_{1\hbar }(ƻ),\vartheta_{2\hbar }(ƻ)\right)$ converges to *ϑ*(ƻ) as ℏ→∞.

**Theorem 4.** An $\varepsilon_{\hbar }(ƻ)=\vartheta (ƻ)-\vartheta_{\hbar }(ƻ)$ can approximate the solution of (1) and (2) by the SGLA.

**Proof.** By leveraging the SGLA, *ϑ*_ℏ_(ƻ) fulfills (1). Hence,

$$\left\{ \begin{array}{l} {\;}^{H}D_{0^{+}}^{\alpha ,\beta }\vartheta_{\hbar }(ƻ)=\left[\begin{array}{c} {\;}^{H}D_{0^{+}}^{\alpha ,\beta }\vartheta_{1\hbar }(ƻ)\\ {\;}^{H}D_{0^{+}}^{\alpha ,\beta }\vartheta_{2\hbar }(ƻ) \end{array}\right]=\left[\begin{array}{c} S_{1\hbar }(ƻ,{℘}_{1\hbar }(\vartheta_{\hbar }(ƻ)),\vartheta_{1\hbar }(ƻ-\sigma_{1}))\\ S_{2\hbar }(ƻ,{℘}_{2\hbar }(\vartheta_{\hbar }(ƻ)),\vartheta_{2\hbar }(ƻ-\sigma_{2})) \end{array}\right],0<ƻ\leq F,\\ \vartheta_{\hbar }(ƻ)=\left[\begin{array}{c} \phi_{1}(ƻ)\\ \phi_{2}(ƻ) \end{array}\right],-\sigma_{1}\leq ƻ\leq 0. \end{array}\right.$$ (67)

Now, by subtracting (67) from (1), one has

$$\left\{ \begin{array}{l} {\;}^{H}D_{0^{+}}^{\alpha ,\beta }\varepsilon_{\hbar }(ƻ)=h(ƻ,\varepsilon_{\hbar }(ƻ),\varepsilon_{\hbar }(ƻ-\theta )),0<ƻ\leq F,\\ \varepsilon_{\hbar }(ƻ)=0,-\sigma_{1}\leq ƻ\leq 0, \end{array}\right.$$ (68)

$$h(ƻ,\varepsilon_{\hbar }(ƻ),\varepsilon_{\hbar }(ƻ-\tau ))=\left[\begin{array}{l} S_{1\hbar }(ƻ,{℘}_{1\hbar }(\vartheta_{\hbar }(ƻ)),\vartheta_{1\hbar }(ƻ-\sigma_{1}))-S_{1}(ƻ,{℘}_{1}(\vartheta (ƻ)),\vartheta_{1}(ƻ-\sigma_{1}))\\ S_{2\hbar }(ƻ,{℘}_{2\hbar }(\vartheta_{\hbar }(ƻ)),\vartheta_{2\hbar }(ƻ-\sigma_{2}))-S_{2}(ƻ,{℘}_{2}(\vartheta (ƻ)),\vartheta_{2}(ƻ-\sigma_{2})) \end{array}\right]$$ (69)

Thus, to solve (68) and (69), we can use SGLA to gain a set of 2(ℏ+1) algebraic equations, and by handling this set one gets *ε*_ℏ_(ƻ).

## 5 Computational demonstrations

Herein, we solve two systems of DFDEs in the FHD sense that involve various instances by leveraging the proposed algorithm. While the second example is nonlinear, the first one is linear. Our methodology involves controlling the relative error $R_{\hbar }(ƻ)=(R_{\hbar ,1}(ƻ),R_{\hbar ,2}(ƻ))$ and the absolute error $A_{\hbar }(ƻ)=(A_{\hbar ,1}(ƻ),A_{\hbar ,2}(ƻ))$ for various values of *α*, *β*, and ℏ. We provide a thorough examination of the effectiveness through plots and data that contrast the exact solution with the approximations.

### 5.1 SGLA: Steps and examples

The SGLA provides several benefits and utilities when handling a set of FDEs as

- The simulated outcomes achieved closely approximates the exact solution. As evidenced by the obtained ℛ_ℏ_(ƻ) and $A_{\hbar }(ƻ)$, SGLA delivers highly sufficient and accurate results.
- The SGLA can achieve high validity by leveraging tiny iterations in the OSLP expansions.
- The SGLA represents a straightforward scheme to incorporate that does not require sophisticated mathematical apparatuses or a proficient programmer.
- The utilized SGLA serves as a universal technique that can be implemented to exhibit other instances of fractional systems.
- The core characteristic of SGLA is its applicability to other orthogonal basis functions.

Algorithm 1 clearly outlines the steps to derive a solution leveraging the presented SGLA and the specified FHD. At this point, either a programmer or a specialized mathematician with relevant expertise could implement this algorithm into a program by leveraging the MATHEMATICA 11 environment, consistent with how we demonstrated this approach in this paper.

**Algorithm 1.** The SGLA iteration steps for calculations of *ϑ*_ℏ_(*x*) concerning DFDE (1) and (2) in the FHD sense.

Requirement:

Primary parameters: *α*,*β*,*τ*, and *σ*;

Nonhomogenous terms: $S_{1}$ and $S_{2}$;

OSLPs of ${\;}^{H}D_{0^{+}}^{\alpha ,\beta }\vartheta_{1}(ƻ)$;

Magnitude of accuracy: ℏ.

Action A:

While ϐ = 0,1,⋯,ℏ find *R*(ƻ) from (62).

Action B:

While ϐ = 1,2,⋯,ℏ−1 find

$\left\{ \begin{array}{l} \int_{0}^{1}‍R_{1}(ƻ)\phi_{ϐ}(ƻ)dƻ=0,\\ \sum_{ϐ=0}^{\hbar }‍a_{ϐ}\phi_{ϐ}(0)=\phi_{1}(0). \end{array}\right.$

$\left\{ \begin{array}{l} \int_{0}^{1}‍R_{2}(ƻ)\phi_{ϐ}(ƻ)dƻ=0,\\ \sum_{ϐ=0}^{\hbar }‍b_{ϐ}\phi_{ϐ}(0)=\phi_{2}(0). \end{array}\right.$

Action C:

While ϐ = 0,1,⋯,ℏ find $\sum_{ϐ=0}^{\hbar }a_{ϐ}{\left(-1\right)}^{ϐ}$ and $\sum_{ϐ=0}^{\hbar }b_{ϐ}{\left(-1\right)}^{ϐ}$.

Action D:

Solve 2(ℏ+1) set of equations generated from Action B to find $c_{ϐ},ϐ=0,1,\cdots ,\hbar$.

Result:

Assign $a_{ϐ}$ and $b_{ϐ}$ with ϐ = 0,1,⋯,ℏ on OSLP of *ϑ*(ƻ) to get *ϑ*_*ℏ*_(*x*).

Let us begin our calculations with the following two suitable instances, both of which have precise solutions throughout the chosen integral domain, ensuring the accuracy of the data outputs.

**Example 1.** Examine the fraction approximations of the succeeding linear sets of DFDEs in the FHD sense:

$$\left\{ \begin{array}{l} {\;}^{H}D_{0^{+}}^{\alpha ,\beta }\vartheta_{1}(ƻ)=\vartheta_{1}(ƻ-0.25)+\vartheta_{2}(ƻ)+u_{1}(ƻ),0<ƻ\leq 2,\\ {\;}^{H}D_{0^{+}}^{\alpha ,\beta }\vartheta_{2}(ƻ)=\vartheta_{2}(ƻ-1)+\vartheta_{2}(ƻ)+\vartheta_{1}(ƻ)+u_{2}(ƻ),\\ u_{1}(ƻ)=-(ƻ-0.25)-{ƻ}^{2}+\frac{1}{\Gamma (2-\alpha )}{ƻ}^{1-\alpha },\\ u_{2}(ƻ)=-(ƻ-1)-{ƻ}^{2}-ƻ+\frac{2}{\Gamma (3-\alpha )}{ƻ}^{2-\alpha },\\ \vartheta_{1}(ƻ)=ƻ,-0.25\leq ƻ\leq 0,\\ \vartheta_{2}(ƻ)={ƻ}^{2},-1\leq ƻ\leq 0. \end{array}\right.$$ (70)

Hither, *ϑ*_1_(ƻ) = ƻ over ƻ∈[−0.25,2] and *ϑ*_1_(ƻ) = ƻ^2^ over ƻ∈[−1,2]. Indded, 0<*α*<1 and 0<*β*<1.

**Example 2.** Examine the fraction approximations of the succeeding nonlinear sets of DFDEs in the FHD sense:

$$\left\{ \begin{array}{l} {\;}^{H}D_{0^{+}}^{\alpha ,\beta }\vartheta_{1}(ƻ)+\vartheta_{1}(ƻ-1)+{\vartheta_{1}(ƻ)\vartheta }_{2}(ƻ)=u_{1}(ƻ),0<ƻ\leq 2,\\ {\;}^{H}D_{0^{+}}^{\alpha ,\beta }\vartheta_{2}(ƻ)+\vartheta_{2}(ƻ-0.7)+\vartheta_{1}(ƻ)\vartheta_{2}(ƻ)+{\vartheta_{1}}^{2}(ƻ)=u_{2}(ƻ),\\ u_{1}(ƻ)=(ƻ-1)+{ƻ}^{3}+\frac{1}{\Gamma (2-\alpha )}{ƻ}^{1-\alpha },\\ u_{2}(ƻ)=(ƻ-0.7)+{ƻ}^{2}+{ƻ}^{3}+\frac{2}{\Gamma (3-\alpha )}{ƻ}^{2-\alpha },\\ \vartheta_{1}(ƻ)=ƻ,-1\leq ƻ\leq 0,\\ \vartheta_{2}(ƻ)={ƻ}^{2},-0.7\leq ƻ\leq 0. \end{array}\right.$$ (71)

Hither, *ϑ*_1_(ƻ) = ƻ over ƻ∈[−1,1] and *ϑ*_2_(ƻ) = ƻ^2^ over ƻ∈[−0.7,1]. Indded, 0<*α*<1 and 0<*β*<1.

### 5.2 Analysis of the findings

This study applies the preceding numerical technique to approximate solutions to (1) and (2). Graphical and tabulated outputs are generated for each example scenario under varied configurations of {*α*,*β*} and ℏ parameters. Additionally, computations of $A_{\hbar }(ƻ)$ and ℛ_ℏ_(ƻ) are carried out and compared extensively, to evaluate the efficacy of the SGLA numerical approach.

For Example 1; in Table 1, the correlation between data operations $A_{\hbar ,1}(ƻ)$ and ℛ_ℏ,1_(ƻ) are presented at ℏ = 3, *α* = 0.5, and *β*∈{0.3,0.8}. In Table 2, the correlation between data operations $A_{\hbar ,2}(ƻ)$ and ℛ_ℏ,2_(ƻ) are presented at ℏ = 3, *α* = 0.5, and *β*∈{0.3,0.8}. To provide additional information, Fig 1 displays $A_{\hbar ,1}(ƻ)$ and ℛ_ℏ,1_(ƻ) at (ℏ,α,β)∈{(3,0.3,0.3),(3,0.4,0.9),(3,0.9,0.4)} that generated from the SGLA. Fig 2 displays $A_{\hbar ,2}(ƻ)$ and ℛ_ℏ,2_(ƻ) at (ℏ,α,β)∈{(3,0.3,0.3),(3,0.4,0.9),(3,0.9,0.4)} that generated from the SGLA.

**Fig 1:**
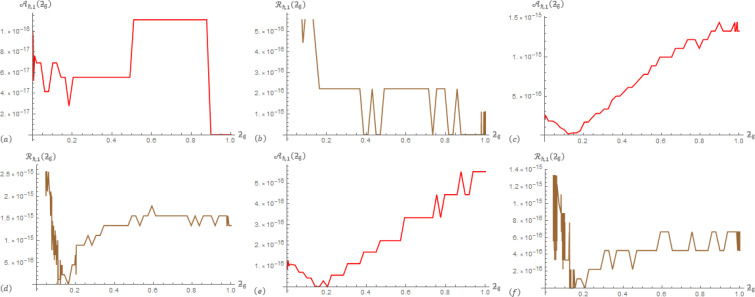
The SGLA depicts the evolution plot of $A_{\hbar ,1}({ƻ}_{ϐ})$ (red) and ℛ_ℏ,1_(ƻ_ϐ_) (brown) with ℏ = 3 over [0,1] in Example 1 at: (a) & (b) (*α*,*β*) = (0.3,0.3), (c) & (d) (*α*,*β*) = (0.4,0.9), and (e) & (f) (*α*,*β*) = (0.9,0.4).

**Fig 2:**
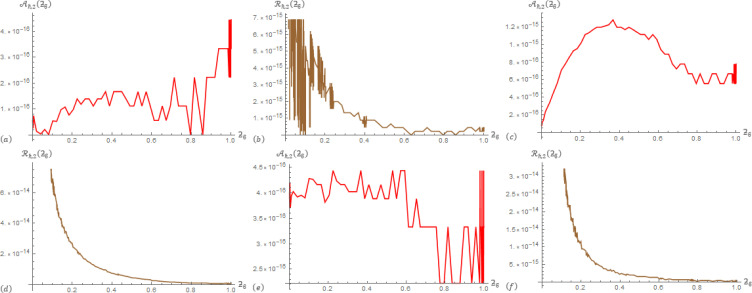
The SGLA depicts the evolution plot of $A_{\hbar ,2}({ƻ}_{ϐ})$ (red) and ℛ_ℏ,2_(ƻ_ϐ_) (brown) with ℏ = 3 over [0,1] in Example 1 at: (a) & (b) (*α*,*β*) = (0.3,0.3), (c) & (d) (*α*,*β*) = (0.4,0.9), and (e) & (f) (*α*,*β*) = (0.9,0.4).

**Table 1:** The SGLA comparison data of $A_{\hbar ,1}(ƻ)$ and ℛ_ℏ,1_(ƻ) with ℏ = 3 at *α* = 0.5 and *β*∈{0.3,0.8} in Example 1.

*β*	0.3		0.8	
ƻ_ϐ_	$A_{\hbar ,1}({ƻ}_{ϐ})$	ℛ_ℏ,1_(ƻ_ϐ_)	$A_{\hbar ,1}({ƻ}_{ϐ})$	ℛ_ℏ,1_(ƻ_ϐ_)
0.1	$9.7144514654\times 10^{-17}$	$8.8817841970\times 10^{-16}$	$1.1102230246\times 10^{-16}$	$1.1102230246\times 10^{-15}$
0.2	$2.7755575615\times 10^{-17}$	$2.2204460492\times 10^{-16}$	$2.2204460492\times 10^{-16}$	$1.1102230246\times 10^{-15}$
0.3	$1.6653345369\times 10^{-16}$	$6.6613381477\times 10^{-16}$	$2.7755575615\times 10^{-16}$	$8.8817841970\times 10^{-16}$
0.4	$2.7755575615\times 10^{-16}$	$6.6613381477\times 10^{-16}$	$3.8857805861\times 10^{-16}$	$1.1102230246\times 10^{-15}$
0.5	$4.4408920985\times 10^{-16}$	$8.8817841970\times 10^{-16}$	$5.5511151231\times 10^{-16}$	$1.1102230246\times 10^{-15}$
0.6	$5.5511151231\times 10^{-16}$	$8.8817841970\times 10^{-16}$	$6.6613381477\times 10^{-16}$	$1.1102230246\times 10^{-15}$
0.7	$8.8817841970\times 10^{-16}$	$1.3322676295\times 10^{-15}$	$7.7715611723\times 10^{-16}$	$1.1102230246\times 10^{-15}$
0.8	$9.9920072216\times 10^{-16}$	$1.3322676295\times 10^{-15}$	$8.8817841970\times 10^{-16}$	$1.1102230246\times 10^{-15}$
0.9	$1.22124532708\times 10^{-15}$	$1.3322676295\times 10^{-15}$	$9.9920072216\times 10^{-16}$	$1.1102230246\times 10^{-15}$
1	$1.3322676295\times 10^{-15}$	$1.3322676295\times 10^{-15}$	$1.1102230246\times 10^{-15}$	$1.1102230246\times 10^{-15}$

**Table 2:** The SGLA comparison data of $A_{\hbar ,2}(ƻ)$ and ℛ_ℏ,2_(ƻ) with ℏ = 3 at *α* = 0.5 and *β*∈{0.3,0.8} in Example 1.

*β*	0.3		0.8	
ƻ_ϐ_	$A_{\hbar ,2}({ƻ}_{ϐ})$	ℛ_ℏ,2_(ƻ_ϐ_)	$A_{\hbar ,2}({ƻ}_{ϐ})$	ℛ_ℏ,2_(ƻ_ϐ_)
0.1	$2.1510571102\times 10^{-16}$	$2.1538326677\times 10^{-14}$	$2.4286128663\times 10^{-16}$	$2.4424906541\times 10^{-14}$
0.2	$4.5102810375\times 10^{-16}$	$1.1324274851\times 10^{-14}$	$4.4408920985\times 10^{-16}$	$1.1102230246\times 10^{-14}$
0.3	$6.8001160258\times 10^{-16}$	$7.5495165674\times 10^{-15}$	$5.8286708792\times 10^{-16}$	$6.4392935428\times 10^{-15}$
0.4	$7.4940054162\times 10^{-16}$	$4.6629367034\times 10^{-15}$	$5.5511151231\times 10^{-16}$	$3.5527136788\times 10^{-15}$
0.5	$8.3266726846\times 10^{-16}$	$3.3306690738\times 10^{-15}$	$6.6613381477\times 10^{-16}$	$2.6645352591\times 10^{-15}$
0.6	$8.881784197\times 10^{-16}$	$2.4424906541\times 10^{-15}$	$7.2164496600\times 10^{-16}$	$1.9984014443\times 10^{-15}$
0.7	$8.8817841970\times 10^{-16}$	$1.9984014443\times 10^{-15}$	$6.6613381477\times 10^{-16}$	$1.5543122344\times 10^{-15}$
0.8	$7.7715611723\times 10^{-16}$	$1.3322676295\times 10^{-15}$	$5.5511151231\times 10^{-16}$	$8.8817841970\times 10^{-16}$
0.9	$8.8817841970\times 10^{-16}$	$1.1102230246\times 10^{-15}$	$5.5511151231\times 10^{-16}$	$6.6613381477\times 10^{-16}$
1	$8.8817841970\times 10^{-16}$	$8.8817841970\times 10^{-16}$	$6.6613381477\times 10^{-16}$	$6.6613381477\times 10^{-16}$

For Example 2; in Table 3, the correlation between data operations $A_{\hbar ,2}(ƻ)$ and ℛ_ℏ,1_(ƻ) are presented at ℏ = 3, *β* = 0.5, and *α*∈{0.1,0.7}. In Table 4, the correlation between data operations $A_{\hbar ,2}(ƻ)$ and ℛ_ℏ,2_(ƻ) are presented at ℏ = 3, *β* = 0.5, and *α*∈{0.1,0.7}. To provide additional information, Fig 3 displays $A_{\hbar ,1}(ƻ)$ and ℛ_ℏ,1_(ƻ) at (ℏ,α,β)∈{(3,0.3,0.6),(3,0.6,0.3),(3,0.4,0.4)} that generated from the SGLA. Fig 4 displays $A_{\hbar ,2}(ƻ)$ and $R_{\hbar ,2}(ƻ)$ at (ℏ,α,β)∈{(3,0.3,0.6),(3,0.6,0.3),(3,0.4,0.4)} that generated from the SGLA.

**Fig 3:**
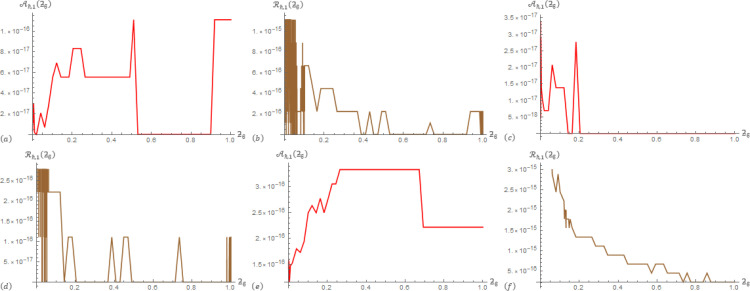
The SGLA depicts the evolution plot of $A_{\hbar ,1}({ƻ}_{ϐ})$ (red) and ℛ_ℏ,1_(ƻ_ϐ_) (brown) with ℏ = 3 over [0,1] in Example 2 at: (a) & (b) (*α*,*β*) = (0.3,0.6), (c) & (d) (*α*,*β*) = (0.6,0.3), and (e) & (f) (*α*,*β*) = (0.4,0.4).

**Fig 4:**
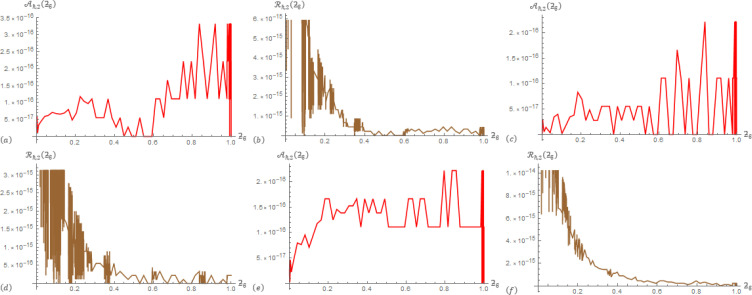
The SGLA depicts the evolution plot of $A_{\hbar ,2}({ƻ}_{ϐ})$ (red) and ℛ_ℏ,2_(ƻ_ϐ_) (brown) with ℏ = 3 over [0,1] in Example 2 at: (a) & (b) (*α*,*β*) = (0.3,0.6), (c) & (d) (*α*,*β*) = (0.6,0.3), and (e) & (f) (*α*,*β*) = (0.4,0.4).

**Table 3:** The SGLA comparison data of $A_{\hbar ,1}(ƻ)$ and ℛ_ℏ,1_(ƻ) with ℏ = 3 at *α*∈{0.1,0.7} and *β* = 0.5 in Example 2.

*α*	0.1		0.7	
ƻ_ϐ_	$A_{\hbar ,1}({ƻ}_{ϐ})$	ℛ_ℏ,1_(ƻ_ϐ_)	$A_{\hbar ,1}({ƻ}_{ϐ})$	ℛ_ℏ,1_(ƻ_ϐ_)
0.1	$1.7624790515\times 10^{-15}$	$1.7541523789\times 10^{-14}$	$8.3266726846\times 10^{-17}$	$7.7715611723\times 10^{-16}$
0.2	$2.0816681711\times 10^{-15}$	$1.0325074129\times 10^{-14}$	$8.3266726846\times 10^{-17}$	$3.3306690738\times 10^{-16}$
0.3	$2.1094237467\times 10^{-15}$	$6.9944050551\times 10^{-15}$	$1.1102230246\times 10^{-16}$	$3.3306690738\times 10^{-16}$
0.4	$1.9984014443\times 10^{-15}$	$4.8849813083\times 10^{-15}$	$1.1102230246\times 10^{-16}$	$2.2204460492\times 10^{-16}$
0.5	$1.6653345369\times 10^{-15}$	$3.3306690738\times 10^{-15}$	$1.1102230246\times 10^{-16}$	$2.2204460492\times 10^{-16}$
0.6	$1.4432899320\times 10^{-15}$	$2.4424906541\times 10^{-15}$	$1.1102230246\times 10^{-16}$	$2.2204460492\times 10^{-16}$
0.7	$1.2212453270\times 10^{-15}$	$1.7763568394\times 10^{-15}$	0	0
0.8	$1.2212453270\times 10^{-15}$	$1.4432899320\times 10^{-15}$	0	0
0.9	$1.4432899320\times 10^{-15}$	$1.5543122344\times 10^{-15}$	$1.1102230246\times 10^{-16}$	0
1	$2.2204460492\times 10^{-15}$	$2.2204460492\times 10^{-15}$	0	0

**Table 4:** The SGLA comparison data of $A_{\hbar ,2}(ƻ)$ and ℛ_ℏ,1_(ƻ) with ℏ = 3 at *α*∈{0.1,0.7} and *β =* 0.5 in Example 2.

*α*	0.1		0.7	
ƻ_ϐ_	$A_{\hbar ,2}({ƻ}_{ϐ})$	ℛ_ℏ,2_(ƻ_ϐ_)	$A_{\hbar ,2}({ƻ}_{ϐ})$	ℛ_ℏ,2_(ƻ_ϐ_)
0.1	$5.6205040621\times 10^{-16}$	$5.6177285046\times 10^{-14}$	$6.2450045135\times 10^{-17}$	$6.2172489379\times 10^{-15}$
0.2	$4.1633363423\times 10^{-16}$	$1.0325074129\times 10^{-14}$	0	0
0.3	$8.3266726846\times 10^{-17}$	$9.9920072216\times 10^{-16}$	$4.1633363423\times 10^{-17}$	$4.4408920985\times 10^{-16}$
0.4	$2.4980018054\times 10^{-16}$	$1.5543122344\times 10^{-15}$	0	0
0.5	$7.7715611723\times 10^{-16}$	$3.1086244689\times 10^{-15}$	$5.5511151231\times 10^{-17}$	$2.2204460492\times 10^{-16}$
0.6	$1.2767564783\times 10^{-15}$	$3.5527136788\times 10^{-15}$	$2.2204460492\times 10^{-16}$	$4.4408920985\times 10^{-16}$
0.7	$1.7208456881\times 10^{-15}$	$3.5527136788\times 10^{-15}$	$1.6653345369\times 10^{-16}$	$4.4408920985\times 10^{-16}$
0.8	$1.7763568394\times 10^{-15}$	$2.8865798640\times 10^{-15}$	$1.1102230246\times 10^{-16}$	$2.2204460492\times 10^{-16}$
0.9	$1.9984014443\times 10^{-15}$	$2.4424906541\times 10^{-15}$	$2.2204460492\times 10^{-16}$	$2.2204460492\times 10^{-16}$
1	$1.9984014443\times 10^{-15}$	$1.9984014443\times 10^{-15}$	$2.2204460492\times 10^{-16}$	$2.2204460492\times 10^{-16}$

From the charts in the antecedent figures, we interestingly that the apparent drawing curves are very low towards the ƻ-axis. This confirms the data of the driving tables. In addition, these curves contain oscillations at their beginning or end, which confirms the occurrence of a delay in the fraction solutions, and also confirms the shape of the previously selected examples.

## 6 Summary of key findings

Throughout the work, we established the EUE concerning a set of DFDEs in the sense of the FHD approach. Our investigation involved examining the properties of the FRD, FCD, and FHD, utilizing their hallmarks to convert our system of DFDEs into an equivalent one of fractional DVIE with the help of the CMT. For numerical analysis, we implemented the SGLA leveraging OSLPs. This algorithm allows us to approximate the solution to the considered system by transforming it into a series of algebraic equations. One of the key advantages of this algorithm is its ability to achieve accurate results with fewer iterations compared to alternative methods. The numerical convergence and error estimates of the approach are examined and to validate the effectiveness and accuracy of our algorithm, we conducted a comprehensive evaluation through various linear and nonlinear numerical applications. The data from these evaluations, accompanied by charts and tables, further validate the strength of our algorithmic approach. Future research directions for our simulations could include applying it to additional types of DFDEs, such as those containing multiple delays or nonlinear terms. The algorithm could also be analyzed for solving DFDEs and fractional delay integral models. Furthermore, this research emphasizes the adaptability of the SGLA, as it can be extended to other complex fractional problems and systems by utilizing alternative orthogonal basis functions like Chebyshev or Fourier polynomials. Additionally, the algorithm may be parallelized to improve computational efficiency.

## List of abbreviations

SGLA: Shifted Galerkin Legendre algorithm
DFDE: Delay fractional differential equation
OSLP: Orthogonal shifted Legendre polynomial
FHD: Fractional Hilfer derivative
FCD: Fractional Caputo derivative
FRD: Fractional Riemann derivative
DVIE: Delay Volterra integral equation
EUE: Existence and uniqueness effect
!-Sol: Unique solution
CMT: Contraction mapping theorem
